# Quantitative trait locus analysis of hybrid pedigrees: variance-components model, inbreeding parameter, and power

**DOI:** 10.1186/1471-2156-8-50

**Published:** 2007-07-26

**Authors:** Gulnara R Svischeva

**Affiliations:** 1 Laboratory of Recombination and Segregation Analysis, Institute of Cytology and Genetics, Siberian Branch of the Russian Academy of Science, Lavrentiev Ave, 10, Novosibirsk, 630090, Russia

## Abstract

**Background:**

For the last years reliable mapping of quantitative trait loci (QTLs) has become feasible through linkage analysis based on the variance-components method. There are now many approaches to the QTL analysis of various types of crosses within one population (breed) as well as crosses between divergent populations (breeds). However, to analyse a complex pedigree with dominance and inbreeding, when the pedigree's founders have an inter-population (hybrid) origin, it is necessary to develop a high-powered method taking into account these features of the pedigree.

**Results:**

We offer a universal approach to QTL analysis of complex pedigrees descended from crosses between outbred parental lines with different QTL allele frequencies. This approach improves the established variance-components method due to the consideration of the genetic effect conditioned by inter-population origin and inbreeding of individuals. To estimate model parameters, namely additive and dominant effects, and the allelic frequencies of the QTL analysed, and also to define the QTL positions on a chromosome with respect to genotyped markers, we used the maximum-likelihood method. To detect linkage between the QTL and the markers we propose statistics with a non-central χ^2^-distribution that provides the possibility to deduce analytical expressions for the power of the method and therefore, to estimate the pedigree's size required for 80% power. The method works for arbitrarily structured pedigrees with dominance and inbreeding.

**Conclusion:**

Our method uses the phenotypic values and the marker information for each individual of the pedigree under observation as initial data and can be valuable for fine mapping purposes. The power of the method is increased if the QTL effects conditioned by inter-population origin and inbreeding are enhanced. Several improvements can be developed to take into account fixed factors affecting trait formation, such as age and sex.

## Background

The wide application of DNA markers scattered along the genome together with the rapid development of statistical methods provides reliable localization of quantitative trait loci (QTLs). There are now many approaches to QTL analysis of various types of crosses within one population (breed) as well as crosses between divergent populations (breeds) [1-5]. One of the most powerful approaches to QTL mapping is the variance-components method. In this method, variability among trait observations from individuals within pedigrees is expressed in terms of the effect caused by an unobservable trait-affecting major locus, of the polygenic effect, and of the residual non-genetic effect [2,3,6-13]. The effect attributable to a locus linked to a marker is a function of the additive and dominance components of variance of the locus, the recombination fraction, and the portion of alleles that are identical by descent (IBD) at the marker for each pair of individuals. The polygenic variance component depends only on the relationship between the relative pair.

If the pedigree analysed comes from a population with an identical distribution of genotypes for all the members of the pedigree and with an identical environmental influence on phenotypes, then the covariance between trait values of related pairs is the weighted sum of the variance components identical for all individuals [10,14-16]. The presence of the marker information makes it possible to separate the variance component caused by the locus linked to the marker from the polygenic variance component, and to test the significance of major locus contribution with respect to trait polymorphism.

Crosses between individuals from divergent populations (breeds) that differ by trait distribution are often used in investigations of traits of livestock breeding, laboratory and domestic animals, and studies of human hereditary diseases. A set of statistical methods for QTL mapping was developed in which initial materials that are backcrosses or the *F*_2_-generation descended from inbred lines were used [17-21]. Recently several studies devoted to the analysis of crosses between outbreed lines has been reported [2,3]. One of these statistical methods, known as the segment mapping method [2], is based on division of the genome of hybrid individuals into segments. Here, genetic covariance of a trait is defined for each segment and depends on the variance of initial breeds and the percentage of genetic material of these breeds in this segment. However, this method does not take into consideration such effects as domination and inbreeding. On the contrary, another method developed in [3] assumes the presence of these effects and allows us to find distinctions in genotype frequencies of the major locus analysed between the crossed breeds. The essence of this method is that the genetic covariance of any two individuals is expressed as a non-linear function of the probability of up to 15 possible identity modes differing by the allele origin of the locus. The disadvantage of this method is its inherent complicated calculations.

The objective of the present study is to present another high-powered theoretical approach to analyse data from crosses between outbreed lines using marker information. This approach is based on the variance-components method that takes into account dominance and inbreeding and uses all the pedigree information available. This study is structured as follows. First, we formulate assumptions about the genetic inter-population nature of the trait so that these assumptions allow us to prove the genetic model chosen and the distribution of phenotypes in the pedigree. Second, we develop a universal way for decomposing variance and covariance into equi-type components, so that weighting factors at these components depend on the degree of relationship and the recombination frequency between the marker and the locus, and can be obtained from joint distribution of IBD-alleles of the QTL and the marker [7]. This ensures that we derive the exact analytical expressions of variance components for different types of relative pairs. Third, we obtain analytical expressions for the power of our method without simulation data. The method is demonstrated by an example of hybrid sibships, which are widely popular in experimental designs.

## Results

### The genetic model

A general explanatory multi-locus model describing the quantitative trait for the *i*th individual of a hybrid pedigree is

*X*_i _= *μ*_i _+ *g*_i _+ *G*_i _+ *e*_i_,

where *μ *is the overall mean, *g *and *G *denote independent effects conditioned by the influence of QTLs (major locus and polygene, respectively), and *e *denotes the environmental effect. However, since the contribution of the major locus to the trait studied has no priorities in relation to other loci listed in the polygene, we will consider a simplified mono-locus model, which could be easily extended to general cases without major difficulties.

For the analysis of crosses between two divergent populations, *P*_1 _and *P*_2_, it is necessary to consider additional assumptions about equi-type distribution of the trait in the parental populations, *P*_1 _and *P*_2_, and in the hybrid pedigree, *P*_1 _× *P*_2_. We assume that QTL contributions to trait formation do not depend on the population origin of the individuals, and that crossed initial populations differ by unequal QTL allele frequencies, *p*_1 _for *P*_1 _and *p*_2 _for *P*_2 _[3,23]. In addition, we assume that the Hardy-Weinberg equilibrium is carried out for the *P*_1 _and *P*_2 _populations.

We consider the QTL analysed to be an autosomal locus with alleles *A *and *B*, and its genotypic values *g*, equal to *d*, *a *and -*a *for heterozygotic and homozygotic individuals of alternative forms, respectively. *AA*_i _(*AB*_i_, or *BB*_i_) denotes that individual *i *has genotype *AA *(*AB*, or *BB*). The distribution of frequencies of the QTL genotypes, *AA*_i_, *AB*_i _and *BB*_i_, for the *i*th inbred hybrid individual can be expressed by allelic frequencies of his (her) father (*p*(*A*_f_), *p*(*B*_f_)) and mother (*p*(*A*_m_), *p*(*B*_m_)):

p(AAi)=p(Af)p(Am)+τ,p(ABi)=p(Af)p(Bm)+p(Bf)p(Am)−2τ,p(BBi)=p(Bf)p(Bm)+τ,
 MathType@MTEF@5@5@+=feaafiart1ev1aaatCvAUfKttLearuWrP9MDH5MBPbIqV92AaeXatLxBI9gBaebbnrfifHhDYfgasaacH8akY=wiFfYdH8Gipec8Eeeu0xXdbba9frFj0=OqFfea0dXdd9vqai=hGuQ8kuc9pgc9s8qqaq=dirpe0xb9q8qiLsFr0=vr0=vr0dc8meaabaqaciaacaGaaeqabaqabeGadaaakeaafaqabeWabaaabaGaemiCaaNaeiikaGIaemyqaeKaemyqae0aaSbaaSqaaiabbMgaPbqabaGccqGGPaqkcqGH9aqpcqWGWbaCcqGGOaakcqWGbbqqdaWgaaWcbaGaeeOzaygabeaakiabcMcaPiabdchaWjabcIcaOiabdgeabnaaBaaaleaacqqGTbqBaeqaaOGaeiykaKIaey4kaSIaeqiXdqNaeiilaWcabaGaemiCaaNaeiikaGIaemyqaeKaemOqai0aaSbaaSqaaiabbMgaPbqabaGccqGGPaqkcqGH9aqpcqWGWbaCcqGGOaakcqWGbbqqdaWgaaWcbaGaeeOzaygabeaakiabcMcaPiabdchaWjabcIcaOiabdkeacnaaBaaaleaacqqGTbqBaeqaaOGaeiykaKIaey4kaSIaemiCaaNaeiikaGIaemOqai0aaSbaaSqaaiabbAgaMbqabaGccqGGPaqkcqWGWbaCcqGGOaakcqWGbbqqdaWgaaWcbaGaeeyBa0gabeaakiabcMcaPiabgkHiTiabikdaYiabes8a0jabcYcaSaqaaiabdchaWjabcIcaOiabdkeacjabdkeacnaaBaaaleaacqqGPbqAaeqaaOGaeiykaKIaeyypa0JaemiCaaNaeiikaGIaemOqai0aaSbaaSqaaiabbAgaMbqabaGccqGGPaqkcqWGWbaCcqGGOaakcqWGcbGqdaWgaaWcbaGaeeyBa0gabeaakiabcMcaPiabgUcaRiabes8a0jabcYcaSaaaaaa@7E3F@

where τ denotes a positive-definite parameter of inbreeding caused by the correlation between the uniting gametes of the inbred individual, and shows the difference in genotype frequencies between non-inbred and inbred homozygous individuals [24]. If the individual is non-inbred, then τ = 0. We have considered various types of inbred crosses differing from each other in structure of inbred loops and represented the derivations of the parameter of inbreeding (see section "Parameter of inbreeding" and Appendix). The allelic frequencies, *p*(*A*_i_) and *p*(*B*_i_), are determined through the distribution of genotypes as:

p(Ai)=p(AAi)+12p(ABi),p(Bi)=p(BBi)+12p(ABi).
 MathType@MTEF@5@5@+=feaafiart1ev1aaatCvAUfKttLearuWrP9MDH5MBPbIqV92AaeXatLxBI9gBaebbnrfifHhDYfgasaacH8akY=wiFfYdH8Gipec8Eeeu0xXdbba9frFj0=OqFfea0dXdd9vqai=hGuQ8kuc9pgc9s8qqaq=dirpe0xb9q8qiLsFr0=vr0=vr0dc8meaabaqaciaacaGaaeqabaqabeGadaaakeaafaqabeGabaaabaGaemiCaaNaeiikaGIaemyqae0aaSbaaSqaaiabbMgaPbqabaGccqGGPaqkcqGH9aqpcqWGWbaCcqGGOaakcqWGbbqqcqWGbbqqdaWgaaWcbaGaeeyAaKgabeaakiabcMcaPiabgUcaRmaaliaabaGaeGymaedabaGaeGOmaidaaiabdchaWjabcIcaOiabdgeabjabdkeacnaaBaaaleaacqqGPbqAaeqaaOGaeiykaKIaeiilaWcabaGaemiCaaNaeiikaGIaemOqai0aaSbaaSqaaiabbMgaPbqabaGccqGGPaqkcqGH9aqpcqWGWbaCcqGGOaakcqWGcbGqcqWGcbGqdaWgaaWcbaGaeeyAaKgabeaakiabcMcaPiabgUcaRmaaliaabaGaeGymaedabaGaeGOmaidaaiabdchaWjabcIcaOiabdgeabjabdkeacnaaBaaaleaacqqGPbqAaeqaaOGaeiykaKIaeiOla4caaaaa@5CA9@

Furthermore, the allelic frequencies of the *i*th hybrid individual can be expressed in terms of the allelic frequencies of the initial populations, *p*_1 _and *p*_2_, and a parameter, ε_i1 _(ε_i2 _= 1-ε_i1_), called portion of "blood" of the population *P*_1 _(*P*_2_) [23]:

*p*(*A*_i_) = ε_i1 _*p*_1 _+ (1-ε_i1_) *p*_2_.

We admit that the trait values of individuals from a hybrid pedigree, as well as from *P*_1 _and *P*_2 _populations, have a multi-normal distribution that is parameterised by an expectation vector and a covariance matrix [25]. If the influence of the environment is identical for all hybrid individuals, then without sacrificing the model generality we can assume that environmental effects for all individuals are random effects distributed by the normal law with identical parameters of distribution, *N *(0, Var_e_) [10].

### Parameter of inbreeding

**Figure 1:**
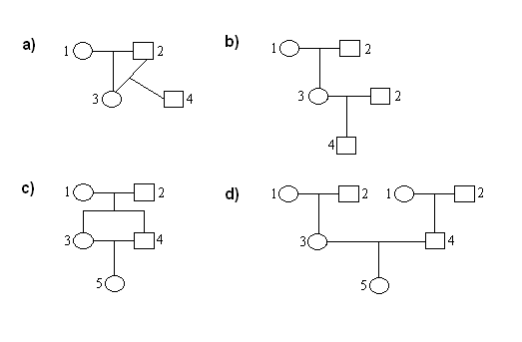
**Pedigrees including the shortest inbred loop with a single and two common ancestors and pedigrees without the loop**. a, b) Pedigrees with and without the loop formed as a result of inbred cross between parent and offspring, respectively. c, d) Pedigrees with and without the loop formed as a result of inbred cross between sibs, respectively.

We have defined the parameter of inbreeding for two particular pedigrees with different inbred loops, and generalized the conclusions drawn to all other pedigrees. Let the first pedigree under review include the shortest inbred loop with a single common ancestor for parents of the inbred individual (Figure 1a). In this case, the inbred individual descends from a cross of the related pair of "parent-offspring". To find τ, we have considered a similar pedigree with the same structure but without inbreeding (Figure 1b). We need to determine the distributions of genotype frequencies for the 4th individuals from inbred and outbred crosses through allelic frequencies of the pedigree's founders, *p*(*A*_1_) and *p*(*A*_2_), and to compare them with each other. For this purpose, we first defined the distribution of genotype frequencies for the 3rd individuals, which are non-inbred for both pedigrees (using formulas (1) at τ = 0):

p(AA3)=p(A1)p(A2),p(AB3)=p(A1)p(B2)+p(B1)p(A2),p(BB3)=p(B1)p(B2).
 MathType@MTEF@5@5@+=feaafiart1ev1aaatCvAUfKttLearuWrP9MDH5MBPbIqV92AaeXatLxBI9gBaebbnrfifHhDYfgasaacH8akY=wiFfYdH8Gipec8Eeeu0xXdbba9frFj0=OqFfea0dXdd9vqai=hGuQ8kuc9pgc9s8qqaq=dirpe0xb9q8qiLsFr0=vr0=vr0dc8meaabaqaciaacaGaaeqabaqabeGadaaakeaafaqaaeWabaaabaGaemiCaaNaeiikaGIaemyqaeKaemyqae0aaSbaaSqaaiabiodaZaqabaGccqGGPaqkcqGH9aqpcqWGWbaCcqGGOaakcqWGbbqqdaWgaaWcbaGaeGymaedabeaakiabcMcaPiabdchaWjabcIcaOiabdgeabnaaBaaaleaacqaIYaGmaeqaaOGaeiykaKIaeiilaWcabaGaemiCaaNaeiikaGIaemyqaeKaemOqai0aaSbaaSqaaiabiodaZaqabaGccqGGPaqkcqGH9aqpcqWGWbaCcqGGOaakcqWGbbqqdaWgaaWcbaGaeGymaedabeaakiabcMcaPiabdchaWjabcIcaOiabdkeacnaaBaaaleaacqaIYaGmaeqaaOGaeiykaKIaey4kaSIaemiCaaNaeiikaGIaemOqai0aaSbaaSqaaiabigdaXaqabaGccqGGPaqkcqWGWbaCcqGGOaakcqWGbbqqdaWgaaWcbaGaeGOmaidabeaakiabcMcaPiabcYcaSaqaaiabdchaWjabcIcaOiabdkeacjabdkeacnaaBaaaleaacqaIZaWmaeqaaOGaeiykaKIaeyypa0JaemiCaaNaeiikaGIaemOqai0aaSbaaSqaaiabigdaXaqabaGccqGGPaqkcqWGWbaCcqGGOaakcqWGcbGqdaWgaaWcbaGaeGOmaidabeaakiabcMcaPiabc6caUaaaaaa@70D9@

As a consequence, allelic frequencies for the 3rd individuals are equal to *p*(*A*_3_) = 1/2 (*p*(*A*_1_) + *p*(*A*_2_)) and *p*(*B*_3_) = 1/2 (*p*(*B*_1_) + *p*(*B*_2_)) in accordance with formulas (2). Therefore we can write the distribution of genotype frequencies for the 4th individual from the outbred cross (Figure 1b):

p(AA4)=12(p(A1)+p(A2))p(A2),p(AB4)=12(p(A1)+p(A2))p(B2)+12(p(B1)+p(B2))p(A2),p(BB4)=12(p(B1)+p(B2))p(B2).
 MathType@MTEF@5@5@+=feaafiart1ev1aaatCvAUfKttLearuWrP9MDH5MBPbIqV92AaeXatLxBI9gBaebbnrfifHhDYfgasaacH8akY=wiFfYdH8Gipec8Eeeu0xXdbba9frFj0=OqFfea0dXdd9vqai=hGuQ8kuc9pgc9s8qqaq=dirpe0xb9q8qiLsFr0=vr0=vr0dc8meaabaqaciaacaGaaeqabaqabeGadaaakeaafaqaaeWabaaabaGaemiCaaNaeiikaGIaemyqaeKaemyqae0aaSbaaSqaaiabisda0aqabaGccqGGPaqkcqGH9aqpdaWccaqaaiabigdaXaqaaiabikdaYaaacqGGOaakcqWGWbaCcqGGOaakcqWGbbqqdaWgaaWcbaGaeGymaedabeaakiabcMcaPiabgUcaRiabdchaWjabcIcaOiabdgeabnaaBaaaleaacqaIYaGmaeqaaOGaeiykaKIaeiykaKIaemiCaaNaeiikaGIaemyqae0aaSbaaSqaaiabikdaYaqabaGccqGGPaqkcqGGSaalaeaacqWGWbaCcqGGOaakcqWGbbqqcqWGcbGqdaWgaaWcbaGaeGinaqdabeaakiabcMcaPiabg2da9maaliaabaGaeGymaedabaGaeGOmaidaaiabcIcaOiabdchaWjabcIcaOiabdgeabnaaBaaaleaacqaIXaqmaeqaaOGaeiykaKIaey4kaSIaemiCaaNaeiikaGIaemyqae0aaSbaaSqaaiabikdaYaqabaGccqGGPaqkcqGGPaqkcqWGWbaCcqGGOaakcqWGcbGqdaWgaaWcbaGaeGOmaidabeaakiabcMcaPiabgUcaRmaaliaabaGaeGymaedabaGaeGOmaidaaiabcIcaOiabdchaWjabcIcaOiabdkeacnaaBaaaleaacqaIXaqmaeqaaOGaeiykaKIaey4kaSIaemiCaaNaeiikaGIaemOqai0aaSbaaSqaaiabikdaYaqabaGccqGGPaqkcqGGPaqkcqWGWbaCcqGGOaakcqWGbbqqdaWgaaWcbaGaeGOmaidabeaakiabcMcaPiabcYcaSaqaaiabdchaWjabcIcaOiabdkeacjabdkeacnaaBaaaleaacqaI0aanaeqaaOGaeiykaKIaeyypa0ZaaSGaaeaacqaIXaqmaeaacqaIYaGmaaGaeiikaGIaemiCaaNaeiikaGIaemOqai0aaSbaaSqaaiabigdaXaqabaGccqGGPaqkcqGHRaWkcqWGWbaCcqGGOaakcqWGcbGqdaWgaaWcbaGaeGOmaidabeaakiabcMcaPiabcMcaPiabdchaWjabcIcaOiabdkeacnaaBaaaleaacqaIYaGmaeqaaOGaeiykaKIaeiOla4caaaaa@983B@

We deal with the conditional distribution of genotype frequencies upon analysing the 4th individual from the inbred cross. Let us therefore fix the genotype of the 2nd individual, *g*_2 _= *AA*, *AB *or *BB *with a probability *p*(*AA*_2_) , *p*(*AB*_2_) or *p*(*BB*_2_). The conditional genotype probabilities for the 3rd and 4th individuals are thus easily calculated and are presented in Tables 1 and 2. According to the formula of total probabilities, the unconditional probability of the genotypes for the 4th inbred individual, *p*^inb^(*g*_4_), is equal to:

**Table 1:** Conditional distribution of genotype frequencies of the QTL for an individual under the given genotype of his (her) parent

*p*(*g*_3_/*g*_2_)	*g*_2 _= *AA*	*g*_2 _= *AB*	*g*_2 _= *BB*
*p*(*AA*_3_/*g*_2_)	*p*(*A*_1_)	1/2 *p*(*A*_1_)	0
*p*(*AB*_3_/*g*_2_)	*p*(*B*_1_)	1/2	*p*(*A*_1_)
*p*(*BB*_3_/*g*_2_)	0	1/2 *p*(*B*_1_)	*p*(*B*_1_)

**Table 2:** Conditional distribution of genotype frequencies of the QTL for an inbred individual originated from cross "parent-offspring" under the given genotype of his (her) parent

*p*(*g*_4_/*g*_2_)	*g*_2 _= *AA*	*g*_2 _= *AB*	*g*_2 _= *BB*	*p*^inb^(*g*_4_)
*p*(*AA*_4_/*g*_2_)	1/2 (*p*(*A*_1_)+1)	1/4(*p*(*A*_1_)+1/2)	0	1/2[*p*(*A*_1_) *p*(*A*_2_) + *p*(*A*_2_) - 1/4*p*(*AB*_2_)]
*p*(*AB*_4_/*g*_2_)	1/2(*p*(*B*_1_)	1/2	1/2*p*(*A*_1_)	1/2[*p*(*B*_1_) *p*(*A*_2_) + *p*(*A*_1_) *p*(*B*_2_) + 1/2*p*(*AB*_2_)]
*p*(*BB*_4_/*g*_2_)	0	1/4(*p*(*B*_1_)+ 1/2)	1/2 (*p*(*B*_1_)+ 1)	1/2[*p*(*B*_1_) *p*(*B*_2_) + *p*(*B*_2_) - 1/4*p*(*AB*_2_)]

pinb(g4)=∑g2p(g2)∑g3p(g4/g3,g2)p(g3/g2)=∑g2p(g4/g2)p(g2).
 MathType@MTEF@5@5@+=feaafiart1ev1aaatCvAUfKttLearuWrP9MDH5MBPbIqV92AaeXatLxBI9gBaebbnrfifHhDYfgasaacH8akY=wiFfYdH8Gipec8Eeeu0xXdbba9frFj0=OqFfea0dXdd9vqai=hGuQ8kuc9pgc9s8qqaq=dirpe0xb9q8qiLsFr0=vr0=vr0dc8meaabaqaciaacaGaaeqabaqabeGadaaakeaafaqabeGabaaabaGaemiCaa3aaWbaaSqabeaacqqGPbqAcqqGUbGBcqqGIbGyaaGccqGGOaakcqWGNbWzdaWgaaWcbaGaeGinaqdabeaakiabcMcaPiabg2da9maaqababaGaemiCaaNaeiikaGIaem4zaC2aaSbaaSqaaiabikdaYaqabaGccqGGPaqkdaaeqaqaaiabdchaWjabcIcaOiabdEgaNnaaBaaaleaacqaI0aanaeqaaOGaei4la8Iaem4zaC2aaSbaaSqaaiabiodaZaqabaGccqGGSaalcqWGNbWzdaWgaaWcbaGaeGOmaidabeaakiabcMcaPiabdchaWjabcIcaOiabdEgaNnaaBaaaleaacqaIZaWmaeqaaOGaei4la8Iaem4zaC2aaSbaaSqaaiabikdaYaqabaGccqGGPaqkaSqaaiabdEgaNnaaBaaameaacqaIZaWmaeqaaaWcbeqdcqGHris5aaWcbaGaem4zaC2aaSbaaWqaaiabikdaYaqabaaaleqaniabggHiLdaakeaacqGH9aqpdaaeqaqaaiabdchaWjabcIcaOiabdEgaNnaaBaaaleaacqaI0aanaeqaaOGaei4la8Iaem4zaC2aaSbaaSqaaiabikdaYaqabaGccqGGPaqkcqWGWbaCcqGGOaakcqWGNbWzdaWgaaWcbaGaeGOmaidabeaakiabcMcaPiabc6caUaWcbaGaem4zaC2aaSbaaWqaaiabikdaYaqabaaaleqaniabggHiLdaaaaaa@7059@

To estimate the parameter of inbreeding, we compared genotype probabilities of the 4th individuals from inbred and outbred crosses, *p*^inb^(*AA*_4_) and *p*(*AA*_4_):

τ=pinb(AA4)−p(AA4)=14(p(A2)p(BB2)+p(B2)p(AA2)).
 MathType@MTEF@5@5@+=feaafiart1ev1aaatCvAUfKttLearuWrP9MDH5MBPbIqV92AaeXatLxBI9gBaebbnrfifHhDYfgasaacH8akY=wiFfYdH8Gipec8Eeeu0xXdbba9frFj0=OqFfea0dXdd9vqai=hGuQ8kuc9pgc9s8qqaq=dirpe0xb9q8qiLsFr0=vr0=vr0dc8meaabaqaciaacaGaaeqabaqabeGadaaakeaafaqadeGabaaabaGaeqiXdqNaeyypa0JaemiCaa3aaWbaaSqabeaacqqGPbqAcqqGUbGBcqqGIbGyaaGccqGGOaakcqWGbbqqcqWGbbqqdaWgaaWcbaGaeGinaqdabeaakiabcMcaPiabgkHiTiabdchaWjabcIcaOiabdgeabjabdgeabnaaBaaaleaacqaI0aanaeqaaOGaeiykaKcabaGaeyypa0ZaaSGaaeaacqaIXaqmaeaacqaI0aanaaGaeiikaGIaemiCaaNaeiikaGIaemyqae0aaSbaaSqaaiabikdaYaqabaGccqGGPaqkcqWGWbaCcqGGOaakcqWGcbGqcqWGcbGqdaWgaaWcbaGaeGOmaidabeaakiabcMcaPiabgUcaRiabdchaWjabcIcaOiabdkeacnaaBaaaleaacqaIYaGmaeqaaOGaeiykaKIaemiCaaNaeiikaGIaemyqaeKaemyqae0aaSbaaSqaaiabikdaYaqabaGccqGGPaqkcqGGPaqkcqGGUaGlaaaaaa@5F35@

The parameter of inbreeding may be generalized to any type of crosses with a single common ancestor:

τ = (1/2)^k ^f(*g*_o_),

where (1/2)^k ^is the degree of relationship of the parents of the inbred individual, and f(*g*_o_) is the function of genotype frequencies of their common ancestor, *g*_o_:

f(*g*_o_) = (*p*(*A*_o_) *p*(*BB*_o_) + *p*(*B*_o_) *p*(*AA*_o_))/2.

The proof of the validity of formulas (3–4) is presented in the Appendix.

Let the second pedigree examined include the shortest inbred loop with two common ancestors of parents of an inbred individual (Figure 1c). In this case, an inbred individual descends from a cross of sibs. To find τ, we considered a similar pedigree with the same structure but without inbreeding (Figure 1d). We need to determine the distributions of the genotype frequencies of the 5th individuals from inbred and outbred crosses through allelic frequencies of the pedigree's founders, *p*(*A*_1_) and *p*(*A*_2_), and to compare them with each other.

At the outbred cross (Figure 1d), genotype frequencies of the 3rd and 4th individuals, *p*(*g*_3_) and *p*(*g*_4_), are identical, and are calculated by formulas (1), when τ = 0. In this case, the 3rd and 4th individuals transfer an allele *A *(*B*) to the offspring with equal probabilities, *p*(*A*_3_) = *p*(*A*_4_) = 1/2 (*p*(*A*_1_) + *p*(*A*_2_)) (*p*(*B*_3_) = *p*(*B*_4_) = 1/2 (*p*(*B*_1_)+ *p*(*B*_2_)). The distribution of genotype frequencies for the 5th individual is thus:

p(AA5)=14(p(A1)+p(A2))2,p(AB5)=12(p(A1)+p(A2))(p(B1)+p(B2)),p(BB5)=14(p(B1)+p(B2))2.
 MathType@MTEF@5@5@+=feaafiart1ev1aaatCvAUfKttLearuWrP9MDH5MBPbIqV92AaeXatLxBI9gBaebbnrfifHhDYfgasaacH8akY=wiFfYdH8Gipec8Eeeu0xXdbba9frFj0=OqFfea0dXdd9vqai=hGuQ8kuc9pgc9s8qqaq=dirpe0xb9q8qiLsFr0=vr0=vr0dc8meaabaqaciaacaGaaeqabaqabeGadaaakeaafaqadeWabaaabaGaemiCaaNaeiikaGIaemyqaeKaemyqae0aaSbaaSqaaiabiwda1aqabaGccqGGPaqkcqGH9aqpdaWccaqaaiabigdaXaqaaiabisda0aaacqGGOaakcqWGWbaCcqGGOaakcqWGbbqqdaWgaaWcbaGaeGymaedabeaakiabcMcaPiabgUcaRiabdchaWjabcIcaOiabdgeabnaaBaaaleaacqaIYaGmaeqaaOGaeiykaKIaeiykaKYaaWbaaSqabeaacqaIYaGmaaGccqGGSaalaeaacqWGWbaCcqGGOaakcqWGbbqqcqWGcbGqdaWgaaWcbaGaeGynaudabeaakiabcMcaPiabg2da9maaliaabaGaeGymaedabaGaeGOmaidaaiabcIcaOiabdchaWjabcIcaOiabdgeabnaaBaaaleaacqaIXaqmaeqaaOGaeiykaKIaey4kaSIaemiCaaNaeiikaGIaemyqae0aaSbaaSqaaiabikdaYaqabaGccqGGPaqkcqGGPaqkcqGGOaakcqWGWbaCcqGGOaakcqWGcbGqdaWgaaWcbaGaeGymaedabeaakiabcMcaPiabgUcaRiabdchaWjabcIcaOiabdkeacnaaBaaaleaacqaIYaGmaeqaaOGaeiykaKIaeiykaKIaeiilaWcabaGaemiCaaNaeiikaGIaemOqaiKaemOqai0aaSbaaSqaaiabiwda1aqabaGccqGGPaqkcqGH9aqpdaWccaqaaiabigdaXaqaaiabisda0aaacqGGOaakcqWGWbaCcqGGOaakcqWGcbGqdaWgaaWcbaGaeGymaedabeaakiabcMcaPiabgUcaRiabdchaWjabcIcaOiabdkeacnaaBaaaleaacqaIYaGmaeqaaOGaeiykaKIaeiykaKYaaWbaaSqabeaacqaIYaGmaaGccqGGUaGlaaaaaa@828C@

At the inbred cross (Figure 1c), the 3rd and 4th individuals as well as their inbred offspring (the 5th individual) therefore have conditional genotype frequencies under fixed genotypes of the 1st and 2nd individuals with the probabilities *p*(*AA*_1_), *p*(*AB*_1_) or *p*(*BB*_1_), and *p*(*AA*_2_), *p*(*AB*_2_) or *p*(*BB*_2_) (Tables 3 and 4). Knowing the conditional genotype frequencies for the 5th individual, we can define unconditional genotype frequencies using the formula of total probabilities:

**Table 3:** Conditional distribution of genotype frequencies of the QTL for sibs under the given genotypes of their parents

*p*(*g*_3,4_/*g*_1_,*g*_2_)	*g*_1 _× *g*_2_					
	
	*AA *× *AA*	*BB *× *BB*	*AB *× *AB*	*BB *× *AA, AA *× *BB*	*AB *× *AA, AA *× *AB*	*BB *× *AB, AB *× *BB*
*p*(*AA*_3,4_/*g*_1_,*g*_2_)	1	0	1/4	0	1/2	0
*p*(*AB*_3,4_/*g*_1_,*g*_2_)	0	0	1/2	1	1/2	1/2
*p*(*BB*_3,4_/*g*_1_,*g*_2_)	0	1	1/4	0	0	1/2

**Table 4:** Conditional distribution of genotype frequencies of the QTL for an inbred individual originated from the cross of sibs under the given genotypes of parents of sibs

*p*(*g*_5_/*g*_1_,*g*_2_)	*g*_1 _× *g*_2_						*p*^inb^(*g*_5_)
		
	*AA *× *AA*	*BB *× *BB*	*AB *× *AB*	*BB *× *AA*, *AA *× *BB*	*AB *× *AA, AA *× *AB*	*BB *× *AB, AB *× *BB*	
*p*(*AA*_5_/*g*_1_,*g*_2_)	1	0	1/4	1/4	9/16	1/16	1/4[*p*(*A*_1_) + *p*(*A*_2_) - 1/4*p*(*AB*_1_) - 1/4*p*(*AB*_2_)+ 2*p*(*A*_1_) *p*(*A*_2_)]
*p*(*AB*_5_/*g*_1_,*g*_2_)	0	0	1/2	1/2	3/8	3/8	1/2[*p*(*B*_1_)*p*(*A*_2_) + *p*(*A*_1_)*p*(*B*_2_) + 1/4*p*(*AB*_1_) + 1/4*p*(*AB*_2_)]
*p*(*BB*_5_/*g*_1_,*g*_2_)	0	1	1/4	1/4	1/16	9/16	1/4[*p*(*B*_1_)+ *p*(*B*_2_)- 1/4*p*(*AB*_1_) - 1/4*p*(*AB*_2_) + 2*p*(*B*_1_) *p*(*B*_2_)]

pinb(g5)=∑g1,g2p(g5/g1,g2)p(g1)p(g2).
 MathType@MTEF@5@5@+=feaafiart1ev1aaatCvAUfKttLearuWrP9MDH5MBPbIqV92AaeXatLxBI9gBaebbnrfifHhDYfgasaacH8akY=wiFfYdH8Gipec8Eeeu0xXdbba9frFj0=OqFfea0dXdd9vqai=hGuQ8kuc9pgc9s8qqaq=dirpe0xb9q8qiLsFr0=vr0=vr0dc8meaabaqaciaacaGaaeqabaqabeGadaaakeaacqWGWbaCdaahaaWcbeqaaiabbMgaPjabb6gaUjabbkgaIbaakiabcIcaOiabdEgaNnaaBaaaleaacqaI1aqnaeqaaOGaeiykaKIaeyypa0ZaaabeaeaacqWGWbaCcqGGOaakcqWGNbWzdaWgaaWcbaGaeGynaudabeaakiabc+caViabdEgaNnaaBaaaleaacqaIXaqmaeqaaOGaeiilaWIaem4zaC2aaSbaaSqaaiabikdaYaqabaGccqGGPaqkcqWGWbaCcqGGOaakcqWGNbWzdaWgaaWcbaGaeGymaedabeaakiabcMcaPiabdchaWjabcIcaOiabdEgaNnaaBaaaleaacqaIYaGmaeqaaOGaeiykaKIaeiOla4caleaacqWGNbWzdaWgaaadbaGaeGymaedabeaaliabcYcaSiabdEgaNnaaBaaameaacqaIYaGmaeqaaaWcbeqdcqGHris5aaaa@57CB@

The parameter of inbreeding is then equal to:

τ=pinb(AA5)−p(AA5)=18(p(A1)p(BB1)+p(B1)p(AA1)+p(A2)p(BB2)+p(B2)p(AA2)),
 MathType@MTEF@5@5@+=feaafiart1ev1aaatCvAUfKttLearuWrP9MDH5MBPbIqV92AaeXatLxBI9gBaebbnrfifHhDYfgasaacH8akY=wiFfYdH8Gipec8Eeeu0xXdbba9frFj0=OqFfea0dXdd9vqai=hGuQ8kuc9pgc9s8qqaq=dirpe0xb9q8qiLsFr0=vr0=vr0dc8meaabaqaciaacaGaaeqabaqabeGadaaakeaafaqadeGabaaabaGaeqiXdqNaeyypa0JaemiCaa3aaWbaaSqabeaacqqGPbqAcqqGUbGBcqqGIbGyaaGccqGGOaakcqWGbbqqcqWGbbqqdaWgaaWcbaGaeGynaudabeaakiabcMcaPiabgkHiTiabdchaWjabcIcaOiabdgeabjabdgeabnaaBaaaleaacqaI1aqnaeqaaOGaeiykaKcabaGaeyypa0ZaaSGaaeaacqaIXaqmaeaacqaI4aaoaaGaeiikaGIaemiCaaNaeiikaGIaemyqae0aaSbaaSqaaiabigdaXaqabaGccqGGPaqkcqWGWbaCcqGGOaakcqWGcbGqcqWGcbGqdaWgaaWcbaGaeGymaedabeaakiabcMcaPiabgUcaRiabdchaWjabcIcaOiabdkeacnaaBaaaleaacqaIXaqmaeqaaOGaeiykaKIaemiCaaNaeiikaGIaemyqaeKaemyqae0aaSbaaSqaaiabigdaXaqabaGccqGGPaqkcqGHRaWkcqWGWbaCcqGGOaakcqWGbbqqdaWgaaWcbaGaeGOmaidabeaakiabcMcaPiabdchaWjabcIcaOiabdkeacjabdkeacnaaBaaaleaacqaIYaGmaeqaaOGaeiykaKIaey4kaSIaemiCaaNaeiikaGIaemOqai0aaSbaaSqaaiabikdaYaqabaGccqGGPaqkcqWGWbaCcqGGOaakcqWGbbqqcqWGbbqqdaWgaaWcbaGaeGOmaidabeaakiabcMcaPiabcMcaPiabcYcaSaaaaaa@784D@

and can be generalized to all inbred individuals having parents with two common ancestors as:

τ = (1/2)^k ^(f(*g*_1o_) + f(*g*_2o_))/2,

where (1/2)^k ^is the degree of relationship of the parents of the inbred individual, and f(*g*_io_) for *i *= 1,2 is the function of genotype frequencies, *g*_io_, of their *i*th common ancestor calculated by formula (4). The proof of the validity of formula (5) is presented in the Appendix.

### Partitioning genetic covariance into components

For the QTL effect, we deduce the formulas of expectation, E_QTL _= *a p*(*AA*_i_) + *d p*(*AB*_i_) - *a p*(*BB*_i_), and variance, Var_QTL _= *a*^2 ^*p*(*AA*_i_) + *d*^2 ^*p*(*AB*_i_) + *a*^2 ^*p*(*BB*_i_) - E_QTL_^2^, depending on the set of parameters {*a*, *d*, *p*(*A*_f_), *p*(*A*_m_), and τ}:

EQTL=12(a+d)(1−2 p(Bf)p(Bm))−12(a−d)(1−2 p(Af)p(Am))−2dτ,VarQTL=p(Af)p(Bf)((a+d)p(Bm)+(a−d)p(Am))2+p(Am)p(Bm)((a−d)p(Af)+(a+d)p(Bf))2+4 p(Af)p(Bf)p(Am)p(Bm)d2+2τ[14((a−d)(p(Af)+p(Am))+(a+d)(p(Bf)+p(Bm)))2+d2(p(Af)−p(Am))2]−4d2τ2.
 MathType@MTEF@5@5@+=feaafiart1ev1aaatCvAUfKttLearuWrP9MDH5MBPbIqV92AaeXatLxBI9gBaebbnrfifHhDYfgasaacH8akY=wiFfYdH8Gipec8Eeeu0xXdbba9frFj0=OqFfea0dXdd9vqai=hGuQ8kuc9pgc9s8qqaq=dirpe0xb9q8qiLsFr0=vr0=vr0dc8meaabaqaciaacaGaaeqabaqabeGadaaakeaafaqabeabbaaaaeaacqqGfbqrdaWgaaWcbaGaeeyuaeLaeeivaqLaeeitaWeabeaakiabg2da9maaliaabaGaeGymaedabaGaeGOmaidaaiabcIcaOiabdggaHjabgUcaRiabdsgaKjabcMcaPiabcIcaOiabigdaXiabgkHiTiabikdaYiabbccaGiabdchaWjabcIcaOiabdkeacnaaBaaaleaacqqGMbGzaeqaaOGaeiykaKIaemiCaaNaeiikaGIaemOqai0aaSbaaSqaaiabb2gaTbqabaGccqGGPaqkcqGGPaqkcqGHsisldaWccaqaaiabigdaXaqaaiabikdaYaaacqGGOaakcqWGHbqycqGHsislcqWGKbazcqGGPaqkcqGGOaakcqaIXaqmcqGHsislcqaIYaGmcqqGGaaicqWGWbaCcqGGOaakcqWGbbqqdaWgaaWcbaGaeeOzaygabeaakiabcMcaPiabdchaWjabcIcaOiabdgeabnaaBaaaleaacqqGTbqBaeqaaOGaeiykaKIaeiykaKIaeyOeI0IaeGOmaiJaemizaqMaeqiXdqNaeiilaWcabaGaeeOvayLaeeyyaeMaeeOCai3aaSbaaSqaaiabbgfarjabbsfaujabbYeambqabaGccqGH9aqpcqWGWbaCcqGGOaakcqWGbbqqdaWgaaWcbaGaeeOzaygabeaakiabcMcaPiabdchaWjabcIcaOiabdkeacnaaBaaaleaacqqGMbGzaeqaaOGaeiykaKIaeiikaGIaeiikaGIaemyyaeMaey4kaSIaemizaqMaeiykaKIaemiCaaNaeiikaGIaemOqai0aaSbaaSqaaiabb2gaTbqabaGccqGGPaqkcqGHRaWkcqGGOaakcqWGHbqycqGHsislcqWGKbazcqGGPaqkcqWGWbaCcqGGOaakcqWGbbqqdaWgaaWcbaGaeeyBa0gabeaakiabcMcaPiabcMcaPmaaCaaaleqabaGaeGOmaidaaaGcbaGaey4kaSIaemiCaaNaeiikaGIaemyqae0aaSbaaSqaaiabb2gaTbqabaGccqGGPaqkcqWGWbaCcqGGOaakcqWGcbGqdaWgaaWcbaGaeeyBa0gabeaakiabcMcaPiabcIcaOiabcIcaOiabdggaHjabgkHiTiabdsgaKjabcMcaPiabdchaWjabcIcaOiabdgeabnaaBaaaleaacqqGMbGzaeqaaOGaeiykaKIaey4kaSIaeiikaGIaemyyaeMaey4kaSIaemizaqMaeiykaKIaemiCaaNaeiikaGIaemOqai0aaSbaaSqaaiabbAgaMbqabaGccqGGPaqkcqGGPaqkdaahaaWcbeqaaiabikdaYaaakiabgUcaRiabisda0iabbccaGiabdchaWjabcIcaOiabdgeabnaaBaaaleaacqqGMbGzaeqaaOGaeiykaKIaemiCaaNaeiikaGIaemOqai0aaSbaaSqaaiabbAgaMbqabaGccqGGPaqkcqWGWbaCcqGGOaakcqWGbbqqdaWgaaWcbaGaeeyBa0gabeaakiabcMcaPiabdchaWjabcIcaOiabdkeacnaaBaaaleaacqqGTbqBaeqaaOGaeiykaKIaemizaq2aaWbaaSqabeaacqaIYaGmaaaakeaacqGHRaWkcqaIYaGmcqaHepaDcqGGBbWwdaWccaqaaiabigdaXaqaaiabisda0aaacqGGOaakcqGGOaakcqWGHbqycqGHsislcqWGKbazcqGGPaqkcqGGOaakcqWGWbaCcqGGOaakcqWGbbqqdaWgaaWcbaGaeeOzaygabeaakiabcMcaPiabgUcaRiabdchaWjabcIcaOiabdgeabnaaBaaaleaacqqGTbqBaeqaaOGaeiykaKIaeiykaKIaey4kaSIaeiikaGIaemyyaeMaey4kaSIaemizaqMaeiykaKIaeiikaGIaemiCaaNaeiikaGIaemOqai0aaSbaaSqaaiabbAgaMbqabaGccqGGPaqkcqGHRaWkcqWGWbaCcqGGOaakcqWGcbGqdaWgaaWcbaGaeeyBa0gabeaakiabcMcaPiabcMcaPiabcMcaPmaaCaaaleqabaGaeGOmaidaaOGaey4kaSIaemizaq2aaWbaaSqabeaacqaIYaGmaaGccqGGOaakcqWGWbaCcqGGOaakcqWGbbqqdaWgaaWcbaGaeeOzaygabeaakiabcMcaPiabgkHiTiabdchaWjabcIcaOiabdgeabnaaBaaaleaacqqGTbqBaeqaaOGaeiykaKIaeiykaKYaaWbaaSqabeaacqaIYaGmaaGccqGGDbqxcqGHsislcqaI0aancqWGKbazdaahaaWcbeqaaiabikdaYaaakiabes8a0naaCaaaleqabaGaeGOmaidaaOGaeiOla4caaaaa@2577@

To partition the genetic variance into components, we modernized the approach of Amos and Elston [7] adapting it for hybrid pedigree analyses.

Let *Y*_j _= (*X*_1j_-*X*_2j_)^2^, where *X*_1j _and *X*_2j _are phenotypic values of a trait measured on individuals 1 and 2 of the *j*th related pair. We denote the proportion (0, 1/2 or 1) of the alleles identical by descent at the QTL and the marker as π_QTLj _and π_Mj_, respectively. Let h⌢Mkj
 MathType@MTEF@5@5@+=feaafiart1ev1aaatCvAUfKttLearuWrP9MDH5MBPbIqV92AaeXatLxBI9gBaebbnrfifHhDYfgasaacH8akY=wiFfYdH8Gipec8Eeeu0xXdbba9frFj0=OqFfea0dXdd9vqai=hGuQ8kuc9pgc9s8qqaq=dirpe0xb9q8qiLsFr0=vr0=vr0dc8meaabaqaciaacaGaaeqabaqabeGadaaakeaadaGiaaqaaiabbIgaObGaayPiJaWaaSbaaSqaaiabb2eanjabbUgaRjabbQgaQbqabaaaaa@330B@ be the estimated probability that the *j*th pair of individuals shares the *k *IBD-alleles (*k *= 0, 1 or 2) at the marker. Then the π_Mj _can be estimated as π⌢Mj=h⌢M2j+12h⌢M1j
 MathType@MTEF@5@5@+=feaafiart1ev1aaatCvAUfKttLearuWrP9MDH5MBPbIqV92AaeXatLxBI9gBaebbnrfifHhDYfgasaacH8akY=wiFfYdH8Gipec8Eeeu0xXdbba9frFj0=OqFfea0dXdd9vqai=hGuQ8kuc9pgc9s8qqaq=dirpe0xb9q8qiLsFr0=vr0=vr0dc8meaabaqaciaacaGaaeqabaqabeGadaaakeaadaGiaaqaaiabec8aWbGaayPiJaWaaSbaaSqaaiabb2eanjabbQgaQbqabaGccqGH9aqpdaGiaaqaaiabbIgaObGaayPiJaWaaSbaaSqaaiabb2eanjabikdaYiabbQgaQbqabaGccqGHRaWkdaWccaqaaiabigdaXaqaaiabikdaYaaadaGiaaqaaiabbIgaObGaayPiJaWaaSbaaSqaaiabb2eanjabigdaXiabbQgaQbqabaaaaa@41EA@. Amos and Elston [7] have shown that covariance, Cov, can be obtained by:

Cov(X1j,X2j|π⌢Mj)=12[Var(X1j)+Var(X2j)]     −12∑πQTLj[E(Yj|πQTLj)∑πMjPr⁡(πQTLj|πMj)Pr⁡(πMj|π⌢Mj)],
 MathType@MTEF@5@5@+=feaafiart1ev1aaatCvAUfKttLearuWrP9MDH5MBPbIqV92AaeXatLxBI9gBaebbnrfifHhDYfgasaacH8akY=wiFfYdH8Gipec8Eeeu0xXdbba9frFj0=OqFfea0dXdd9vqai=hGuQ8kuc9pgc9s8qqaq=dirpe0xb9q8qiLsFr0=vr0=vr0dc8meaabaqaciaacaGaaeqabaqabeGadaaakeaafaqaaeGabaaabaGaee4qamKaee4Ba8MaeeODayNaeiikaGIaemiwaG1aaSbaaSqaaiabigdaXiabbQgaQbqabaGccqGGSaalcqWGybawdaWgaaWcbaGaeGOmaiJaeeOAaOgabeaakiabcYha8naaIaaabaGaeqiWdahacaGLImcadaWgaaWcbaGaeeyta0KaeeOAaOgabeaakiabcMcaPiabg2da9maaliaabaGaeGymaedabaGaeGOmaidaaiabcUfaBjabbAfawjabbggaHjabbkhaYjabcIcaOiabdIfaynaaBaaaleaacqaIXaqmcqqGQbGAaeqaaOGaeiykaKIaey4kaSIaeeOvayLaeeyyaeMaeeOCaiNaeiikaGIaemiwaG1aaSbaaSqaaiabikdaYiabbQgaQbqabaGccqGGPaqkcqGGDbqxaeGabaa9=laaxMaacaWLjaGaeyOeI0YaaSGaaeaacqaIXaqmaeaacqaIYaGmaaWaaabeaeaacqGGBbWwcqqGfbqrcqGGOaakcqWGzbqwdaWgaaWcbaGaeeOAaOgabeaakiabcYha8jabec8aWnaaBaaaleaacqqGrbqucqqGubavcqqGmbatcqqGQbGAaeqaaOGaeiykaKYaaabeaeaacyGGqbaucqGGYbGCcqGGOaakcqaHapaCdaWgaaWcbaGaeeyuaeLaeeivaqLaeeitaWKaeeOAaOgabeaakiabcYha8jabec8aWnaaBaaaleaacqqGnbqtcqqGQbGAaeqaaOGaeiykaKIagiiuaaLaeiOCaiNaeiikaGIaeqiWda3aaSbaaSqaaiabb2eanjabbQgaQbqabaGccqGG8baFdaGiaaqaaiabec8aWbGaayPiJaWaaSbaaSqaaiabb2eanjabbQgaQbqabaGccqGGPaqkcqGGDbqxcqGGSaalaSqaaiabec8aWnaaBaaameaacqqGnbqtcqqGQbGAaeqaaaWcbeqdcqGHris5aaWcbaGaeqiWda3aaSbaaWqaaiabbgfarjabbsfaujabbYeamjabbQgaQbqabaaaleqaniabggHiLdaaaaaa@A18D@

where the conditional probabilities Pr⁡(πMj|π⌢Mj)
 MathType@MTEF@5@5@+=feaafiart1ev1aaatCvAUfKttLearuWrP9MDH5MBPbIqV92AaeXatLxBI9gBaebbnrfifHhDYfgasaacH8akY=wiFfYdH8Gipec8Eeeu0xXdbba9frFj0=OqFfea0dXdd9vqai=hGuQ8kuc9pgc9s8qqaq=dirpe0xb9q8qiLsFr0=vr0=vr0dc8meaabaqaciaacaGaaeqabaqabeGadaaakeaacyGGqbaucqGGYbGCcqGGOaakcqaHapaCdaWgaaWcbaGaeeyta0KaeeOAaOgabeaakiabcYha8naaIaaabaGaeqiWdahacaGLImcadaWgaaWcbaGaeeyta0KaeeOAaOgabeaakiabcMcaPaaa@3C55@ are calculated from the information given on the marker genotypes, whereas the conditional probabilities Pr(π_QTLj_|π_Mj_) depend on the genetic relationship of the relative pair and the recombination frequency between the QTL and the marker, and have already been specified for many types of relatives [7,26]. To determine E(*Y*_j_|π_QTLj_), we list all 9 possible values of *Y*_j _at various QTL genotypes and define the probabilities Pr(*Y*_j_|π_QTLj_) for π_QTLj _= 0, 1/2 or 1:

E(Yj|πQTLj)∑19[YjPr⁡(Yj|πQTLj)].
 MathType@MTEF@5@5@+=feaafiart1ev1aaatCvAUfKttLearuWrP9MDH5MBPbIqV92AaeXatLxBI9gBaebbnrfifHhDYfgasaacH8akY=wiFfYdH8Gipec8Eeeu0xXdbba9frFj0=OqFfea0dXdd9vqai=hGuQ8kuc9pgc9s8qqaq=dirpe0xb9q8qiLsFr0=vr0=vr0dc8meaabaqaciaacaGaaeqabaqabeGadaaakeaacqqGfbqrcqGGOaakcqWGzbqwdaWgaaWcbaGaeeOAaOgabeaakiabcYha8jabec8aWnaaBaaaleaacqqGrbqucqqGubavcqqGmbatcqqGQbGAaeqaaOGaeiykaKYaaabCaeaacqGGBbWwcqWGzbqwdaWgaaWcbaGaeeOAaOgabeaakiGbccfaqjabckhaYjabcIcaOiabdMfaznaaBaaaleaacqqGQbGAaeqaaOGaeiiFaWNaeqiWda3aaSbaaSqaaiabbgfarjabbsfaujabbYeamjabbQgaQbqabaGccqGGPaqkcqGGDbqxcqGGUaGlaSqaaiabigdaXaqaaiabiMda5aqdcqGHris5aaaa@543B@

Let us review in detail the process of finding genetic covariance and its components using the example of sib-pair as the most often used in studies of hereditary diseases.

### The analysis of sib-pair

The sibs present a special interest for researchers, since they are well known to belong to two-linear relatives. We deduced the formulas for the conditional probabilities Pr(*Y*_j_|π_QTLj_) at π_QTLj _= 0, 1/2 or 1 (Table 5), to derive the expressions of expectations E(*Y*_j_|π_QTLj_) by formula (6):

**Table 5:** Conditional probability distribution of *Y*_j _values for pair of sibs

Genotypes of sib-pair	*Y*_j_	Conditional probability Pr (*Y*_j_|π_QTLj_)		
		
		π_QTLj _= 0	π_QTLj _= 1/2	π_QTLj _= 1
*AA-AA*	ξ_j_^2^	*p*(*AA*_f_)*p*(*AA*_m_) + τ	1/2 (*p*(*AA*_f_)*p*(*A*_m_) + *p*(*A*_f_)*p*(*AA*_m_)) + τ	*p*(*A*_f_) *p*(*A*_m_) + τ
*BB-BB*	ξ_j_^2^	*P*(*BB*_f_)*p*(*BB*_m_) +τ	1/2 (*p*(*BB*_f_)*p*(*B*_m_) + *p*(*B*_f_)*p*(*BB*_m_))+τ	*p*(*B*_f_)*p*(*B*_m_)+ τ
*AB-AB*	ξ_j_^2^	*p*(*AA*_f_)*p*(*BB*_m_) + *p*(*BB*_f_)*p*(*AA*_m_) + 1/2*p*(*AB*_f_) *p*(*AB*_m_) -2τ	*p*(*B*_f_)*p*(*A*_m_) + *p*(*A*_f_)*p*(*B*_m_) - 1/4 [*p*(*AB*_f_)+ *p*(*AB*_m_)] - 2 τ	*p*(*A*_f_)*p*(*B*_m_) + *p*(*B*_f_)*p*(*A*_m_)-2τ
*AA-AB*	(*a-d *+ξ_j_)^2^	1/2 (*p*(*AB*_f_)*p*(*AA*_m_) + *p*(*AA*_f_)*p*(*AB*_m_))	1/4(*p*(*A*_f_)*p*(*AB*_m_) + *p*(*AB*_f_)*p*(*A*_m_))	0
*AB-AA*	(-*a *+ *d *+ ξ_j_)^2^	1/2 (*p*(*AB*_f_)*p*(*AA*_m_) + *p*(*AA*_f_)*p*(*AB*_m_))	1/4(*p*(*A*_f_)*p*(*AB*_m_)+ *p*(*AB*_f_)*p*(*A*_m_))	0
*AB-BB*	(*a *+ *d *+ξ_j_)^2^	1/2 (*p*(*AB*_f_)*p*(*BB*_m_) + *p*(*BB*_f_)*p*(*AB*_m_))	1/4(*p*(*B*_f_)*p*(*AB*_m_) + *p*(*AB*_f_)*p*(*B*_m_))	0
*BB-AB*	(-*a-d *+ξ_j_)^2^	1/2 (*p*(*AB*_f_)*p*(*BB*_m_) + *p*(*BB*_f_)*p*(*AB*_m_))	1/4(*p*(*B*_f_)*p*(*AB*_m_) + *p*(*AB*_f_)*p*(*B*_m_))	0
*AA-BB*	(2*a *+ξ_j_)^2^	1/4 *p*(*AB*_f_) *p*(*AB*_m_)	0	0
*BB-AA*	(-2*a *+ ξ_j_)^2^	1/4 *p*(*AB*_f_) *p*(*AB*_m_)	0	0

Total		1	1	1

E(Yj|πQTLj=1)=ξj 2,E(Yj|πQTLj=12)=12(a−d)2(p(Af)p(ABm)+p(ABf)p(Am))     +12(a+d)2(p(Bf)p(ABm)+p(ABf)p(Bm))+ξj 2,E(Yj|πQTLj=0)=(a−d)2p(Af)p(ABm)+p(ABf)p(Am))     +(a+d)2(p(Bf)p(ABm)+p(ABf)p(Bm))−2d2p(ABf)p(ABm)+ξj 2,
 MathType@MTEF@5@5@+=feaafiart1ev1aaatCvAUfKttLearuWrP9MDH5MBPbIqV92AaeXatLxBI9gBamXvP5wqSXMqHnxAJn0BKvguHDwzZbqegyvzYrwyUfgarqqtubsr4rNCHbGeaGqiA8vkIkVAFgIELiFeLkFeLk=iY=Hhbbf9v8qqaqFr0xc9pk0xbba9q8WqFfeaY=biLkVcLq=JHqVepeea0=as0db9vqpepesP0xe9Fve9Fve9GapdbaqaaeGacaGaaiaabeqaamqadiabaaGcbaqbaeaabuqaaaaabaGaeeyrauKaeiikaGIaemywaK1aaSbaaSqaaiabbQgaQbqabaGccqGG8baFcqaHapaCdaWgaaWcbaGaeeyuaeLaeeivaqLaeeitaWKaeeOAaOgabeaakiabg2da9iabigdaXiabcMcaPiabg2da9iabe67a4naaDaaaleaacqqGQbGAaeaacqqGGaaicqqGYaGmaaGccqGGSaalaeaacqqGfbqrcqGGOaakcqWGzbqwdaWgaaWcbaGaeeOAaOgabeaakiabcYha8jabec8aWnaaBaaaleaacqqGrbqucqqGubavcqqGmbatcqqGQbGAaeqaaOGaeyypa0ZaaSGaaeaacqaIXaqmaeaacqaIYaGmaaGaeiykaKIaeyypa0ZaaSGaaeaacqaIXaqmaeaacqaIYaGmaaGaeiikaGIaemyyaeMaeyOeI0IaemizaqMaeiykaKYaaWbaaSqabeaacqaIYaGmaaGccqGGOaakcqWGWbaCcqGGOaakcqWGbbqqdaWgaaWcbaGaeeOzaygabeaakiabcMcaPiabdchaWjabcIcaOiabdgeabjabdkeacnaaBaaaleaacqqGTbqBaeqaaOGaeiykaKIaey4kaSIaemiCaaNaeiikaGIaemyqaeKaemOqai0aaSbaaSqaaiabbAgaMbqabaGccqGGPaqkcqWGWbaCcqGGOaakcqWGbbqqdaWgaaWcbaGaeeyBa0gabeaakiabcMcaPiabcMcaPaqacOaa8aaaCSaa8TaaOVaaGXaaOYaaG0aaO4aa0bba0hHaaCzcaiaaxMaacaWLjaGaaCzcaiabgUcaRmaaliaabaGaeGymaedabaGaeGOmaidaaiabcIcaOiabdggaHjabgUcaRiabdsgaKjabcMcaPmaaCaaaleqabaGaeGOmaidaaOGaeiikaGIaemiCaaNaeiikaGIaemOqai0aaSbaaSqaaiabbAgaMbqabaGccqGGPaqkcqWGWbaCcqGGOaakcqWGbbqqcqWGcbGqdaWgaaWcbaGaeeyBa0gabeaakiabcMcaPiabgUcaRiabdchaWjabcIcaOiabdgeabjabdkeacnaaBaaaleaacqqGMbGzaeqaaOGaeiykaKIaemiCaaNaeiikaGIaemOqai0aaSbaaSqaaiabb2gaTbqabaGccqGGPaqkcqGGPaqkcqGHRaWkcqaH+oaEdaqhaaWcbaGaeeOAaOgabaGaeeiiaaIaeeOmaidaaOGaeiilaWcabaGaeeyrauKaeiikaGIaemywaK1aaSbaaSqaaiabbQgaQbqabaGccqGG8baFcqaHapaCdaWgaaWcbaGaeeyuaeLaeeivaqLaeeitaWKaeeOAaOgabeaakiabg2da9iabicdaWiabcMcaPiabg2da9iabcIcaOiabdggaHjabgkHiTiabdsgaKjabcMcaPmaaCaaaleqabaGaeGOmaidaaOGaemiCaaNaeiikaGIaemyqae0aaSbaaSqaaiabbAgaMbqabaGccqGGPaqkcqWGWbaCcqGGOaakcqWGbbqqcqWGcbGqdaWgaaWcbaGaeeyBa0gabeaakiabcMcaPiabgUcaRiabdchaWjabcIcaOiabdgeabjabdkeacnaaBaaaleaacqqGMbGzaeqaaOGaeiykaKIaemiCaaNaeiikaGIaemyqae0aaSbaaSqaaiabb2gaTbqabaGccqGGPaqkcqGGPaqkaeGamaajaaaHaaa9laavmaaTmaarnaaGnaazoaaBpaaxqaakraaRriaaxMaacaWLjaGaaCzcaiaaxMaacaWLjaGaey4kaSIaeiikaGIaemyyaeMaey4kaSIaemizaqMaeiykaKYaaWbaaSqabeaacqaIYaGmaaGccqGGOaakcqWGWbaCcqGGOaakcqWGcbGqdaWgaaWcbaGaeeOzaygabeaakiabcMcaPiabdchaWjabcIcaOiabdgeabjabdkeacnaaBaaaleaacqqGTbqBaeqaaOGaeiykaKIaey4kaSIaemiCaaNaeiikaGIaemyqaeKaemOqai0aaSbaaSqaaiabbAgaMbqabaGccqGGPaqkcqWGWbaCcqGGOaakcqWGcbGqdaWgaaWcbaGaeeyBa0gabeaakiabcMcaPiabcMcaPiabgkHiTiabikdaYiabdsgaKnaaCaaaleqabaGaeGOmaidaaOGaemiCaaNaeiikaGIaemyqaeKaemOqai0aaSbaaSqaaiabbAgaMbqabaGccqGGPaqkcqWGWbaCcqGGOaakcqWGbbqqcqWGcbGqdaWgaaWcbaGaeeyBa0gabeaakiabcMcaPiabgUcaRiabe67a4naaDaaaleaacqqGQbGAaeaacqqGGaaicqqGYaGmaaGccqGGSaalaaaaaa@32DE@

where ξ_j _is the trait value difference caused by the environment. Apparently, a dependence on the parameter of inbreeding is not present in formulas (7) for E(*Y*_j_|π_QTLj_). This means that the components caused by inbreeding are identical for covariance and variance.

Haseman and Elston [26] have shown that

E(Yj|πQTLj=1)=Z,E(Yj|πQTLj=12)=A+2D+Z,E(Yj|πQTLj=0)=2A+2D+Z,
 MathType@MTEF@5@5@+=feaafiart1ev1aaatCvAUfKttLearuWrP9MDH5MBPbIqV92AaeXatLxBI9gBaebbnrfifHhDYfgasaacH8akY=wiFfYdH8Gipec8Eeeu0xXdbba9frFj0=OqFfea0dXdd9vqai=hGuQ8kuc9pgc9s8qqaq=dirpe0xb9q8qiLsFr0=vr0=vr0dc8meaabaqaciaacaGaaeqabaqabeGadaaakeaafaqaaeWabaaabaGaeeyrauKaeiikaGIaemywaK1aaSbaaSqaaiabbQgaQbqabaGccqGG8baFcqaHapaCdaWgaaWcbaGaeeyuaeLaeeivaqLaeeitaWKaeeOAaOgabeaakiabg2da9iabigdaXiabcMcaPiabg2da9iabbQfaAjabcYcaSaqaaiabbweafjabcIcaOiabdMfaznaaBaaaleaacqqGQbGAaeqaaOGaeiiFaWNaeqiWda3aaSbaaSqaaiabbgfarjabbsfaujabbYeamjabbQgaQbqabaGccqGH9aqpdaWccaqaaiabigdaXaqaaiabikdaYaaacqGGPaqkcqGH9aqpcqqGbbqqcqGHRaWkcqaIYaGmcqqGebarcqGHRaWkcqqGAbGwcqGGSaalaeaacqqGfbqrcqGGOaakcqWGzbqwdaWgaaWcbaGaeeOAaOgabeaakiabcYha8jabec8aWnaaBaaaleaacqqGrbqucqqGubavcqqGmbatcqqGQbGAaeqaaOGaeyypa0JaeGimaaJaeiykaKIaeyypa0JaeGOmaiJaeeyqaeKaey4kaSIaeGOmaiJaeeiraqKaey4kaSIaeeOwaOLaeiilaWcaaaaa@710B@

where Z is the environmental variance component; A and D are additive and dominance variance components, respectively. When the pedigree under consideration is pure (*p *= *p*(*A*_f_) = *p*(*A*_m_) and *q *= 1-*p*), A and D are defined as:

A=2 p q [(a−d)p+(a+d)q]2,D=4 p2 q2 d2.
 MathType@MTEF@5@5@+=feaafiart1ev1aaatCvAUfKttLearuWrP9MDH5MBPbIqV92AaeXatLxBI9gBaebbnrfifHhDYfgasaacH8akY=wiFfYdH8Gipec8Eeeu0xXdbba9frFj0=OqFfea0dXdd9vqai=hGuQ8kuc9pgc9s8qqaq=dirpe0xb9q8qiLsFr0=vr0=vr0dc8meaabaqaciaacaGaaeqabaqabeGadaaakeaafaqaaeGabaaabaGaeeyqaeKaeyypa0JaeGOmaiJaeeiiaaIaemiCaaNaeeiiaaIaemyCaeNaeeiiaaIaei4waSLaeiikaGIaemyyaeMaeyOeI0IaemizaqMaeiykaKIaemiCaaNaey4kaSIaeiikaGIaemyyaeMaey4kaSIaemizaqMaeiykaKIaemyCaeNaeiyxa01aaWbaaSqabeaacqaIYaGmaaGccqGGSaalaeaacqqGebarcqGH9aqpcqaI0aancqqGGaaicqWGWbaCdaahaaWcbeqaaiabikdaYaaakiabbccaGiabdghaXnaaCaaaleqabaGaeGOmaidaaOGaeeiiaaIaemizaq2aaWbaaSqabeaacqaIYaGmaaGccqGGUaGlaaaaaa@5570@

Let us find similar A and D parameters for hybrid sibs. In this case, to determine the covariance between sibs, weighting factors at components A and D are standard functions that depend on the proportion of marker IBD-alleles between sibs and the recombination frequency [10,26]:

f(π⌢Mj,θ)=1−Ψ−(1−2Ψ)π⌢Mj,g(π⌢Mj,θ)=(1−Ψ)2−2(1−Ψ)(1−2Ψ)π⌢Mj+(1−2Ψ)2h⌢M2j,
 MathType@MTEF@5@5@+=feaafiart1ev1aaatCvAUfKttLearuWrP9MDH5MBPbIqV92AaeXatLxBI9gBaebbnrfifHhDYfgasaacH8akY=wiFfYdH8Gipec8Eeeu0xXdbba9frFj0=OqFfea0dXdd9vqai=hGuQ8kuc9pgc9s8qqaq=dirpe0xb9q8qiLsFr0=vr0=vr0dc8meaabaqaciaacaGaaeqabaqabeGadaaakeaafaqaaeGabaaabaacbeGae8NzayMaeiikaGYaaicaaeaacqaHapaCaiaawkYiamaaBaaaleaacqqGnbqtcqqGQbGAaeqaaOGaeiilaWIaeqiUdeNaeiykaKIaeyypa0JaeGymaeJaeyOeI0IaeuiQdKLaeyOeI0IaeiikaGIaeGymaeJaeyOeI0IaeGOmaiJaeuiQdKLaeiykaKYaaicaaeaacqaHapaCaiaawkYiamaaBaaaleaacqqGnbqtcqqGQbGAaeqaaOGaeiilaWcabaGae83zaCMaeiikaGYaaicaaeaacqaHapaCaiaawkYiamaaBaaaleaacqqGnbqtcqqGQbGAaeqaaOGaeiilaWIaeqiUdeNaeiykaKIaeyypa0JaeiikaGIaeGymaeJaeyOeI0IaeuiQdKLaeiykaKYaaWbaaSqabeaacqaIYaGmaaGccqGHsislcqaIYaGmcqGGOaakcqaIXaqmcqGHsislcqqHOoqwcqGGPaqkcqGGOaakcqaIXaqmcqGHsislcqaIYaGmcqqHOoqwcqGGPaqkdaGiaaqaaiabec8aWbGaayPiJaWaaSbaaSqaaiabb2eanjabbQgaQbqabaGccqGHRaWkcqGGOaakcqaIXaqmcqGHsislcqaIYaGmcqqHOoqwcqGGPaqkdaahaaWcbeqaaiabikdaYaaakmaaIaaabaGaeeiAaGgacaGLImcadaWgaaWcbaGaeeyta0KaeGOmaiJaeeOAaOgabeaakiabcYcaSaaaaaa@7D60@

where Ψ = θ^2 ^+ (1-θ)^2^.

By equating expressions (7) and (8) we deduce formulas of A and D for hybrid sibs:

A=p(ABf)[12((a−d)p(Am)+(a+d)p(Bm))2−d2βm]+p(ABm)[12((a−d)p(Af)+(a+d)p(Bf))2−d2βf],D=p(ABf)p(ABm)d2,Z=ξj 2,
 MathType@MTEF@5@5@+=feaafiart1ev1aaatCvAUfKttLearuWrP9MDH5MBPbIqV92AaeXatLxBI9gBamXvP5wqSXMqHnxAJn0BKvguHDwzZbqegyvzYrwyUfgarqqtubsr4rNCHbGeaGqiA8vkIkVAFgIELiFeLkFeLk=iY=Hhbbf9v8qqaqFr0xc9pk0xbba9q8WqFfeaY=biLkVcLq=JHqVepeea0=as0db9vqpepesP0xe9Fve9Fve9GapdbaqaaeGacaGaaiaabeqaamqadiabaaGcbaqbaeaabqqaaaaabaGaeeyqaeKaeyypa0JaemiCaaNaeiikaGIaemyqaeKaemOqai0aaSbaaSqaaiabbAgaMbqabaGccqGGPaqkcqGGBbWwdaWccaqaaiabigdaXaqaaiabikdaYaaacqGGOaakcqGGOaakcqWGHbqycqGHsislcqWGKbazcqGGPaqkcqWGWbaCcqGGOaakcqWGbbqqdaWgaaWcbaGaeeyBa0gabeaakiabcMcaPiabgUcaRiabcIcaOiabdggaHjabgUcaRiabdsgaKjabcMcaPiabdchaWjabcIcaOiabdkeacnaaBaaaleaacqqGTbqBaeqaaOGaeiykaKIaeiykaKYaaWbaaSqabeaacqaIYaGmaaGccqGHsislcqWGKbazdaahaaWcbeqaaiabikdaYaaakiabek7aInaaBaaaleaacqqGTbqBaeqaaOGaeiyxa0fabaGaey4kaSIaemiCaaNaeiikaGIaemyqaeKaemOqai0aaSbaaSqaaiabb2gaTbqabaGccqGGPaqkcqGGBbWwdaWccaqaaiabigdaXaqaaiabikdaYaaacqGGOaakcqGGOaakcqWGHbqycqGHsislcqWGKbazcqGGPaqkcqWGWbaCcqGGOaakcqWGbbqqdaWgaaWcbaGaeeOzaygabeaakiabcMcaPiabgUcaRiabcIcaOiabdggaHjabgUcaRiabdsgaKjabcMcaPiabdchaWjabcIcaOiabdkeacnaaBaaaleaacqqGMbGzaeqaaOGaeiykaKIaeiykaKYaaWbaaSqabeaacqaIYaGmaaGccqGHsislcqWGKbazdaahaaWcbeqaaiabikdaYaaakiabek7aInaaBaaaleaacqqGMbGzaeqaaOGaeiyxa0LaeiilaWcabaGaeeiraqKaeyypa0JaemiCaaNaeiikaGIaemyqaeKaemOqai0aaSbaaSqaaiabbAgaMbqabaGccqGGPaqkcqWGWbaCcqGGOaakcqWGbbqqcqWGcbGqdaWgaaWcbaGaeeyBa0gabeaakiabcMcaPiabdsgaKnaaCaaaleqabaGaeGOmaidaaOGaeiilaWcabaGaeeOwaOLaeyypa0JaeqOVdG3aa0baaSqaaiabbQgaQbqaaiabbccaGiabbkdaYaaakiabcYcaSaaaaaa@B2B3@

where β_p _= (*p*(*AB*_p_) - 2 *p*(*A*_p_) *p*(*B*_p_)) at p = m, f.

The trait variance can be partitioned into components:

Var = A + D + R + Z,

where R is the residual component caused by inter-population origin and inbreeding of sibs):(R = 0 for "pure" non-inbred sibs):

R=d2βfβm−12βm((a−d)p(Af)+(a+d)p(Bf))2−12βf((a−d)p(Am)+(a+d)p(βm))2+2τ[14((a−d)(p(Af)+p(Am))+(a+d)(p(Bf)+p(Bm)))2+d2(p(Af)−p(Am))2]−4d2τ2.
 MathType@MTEF@5@5@+=feaafiart1ev1aaatCvAUfKttLearuWrP9MDH5MBPbIqV92AaeXatLxBI9gBaebbnrfifHhDYfgasaacH8akY=wiFfYdH8Gipec8Eeeu0xXdbba9frFj0=OqFfea0dXdd9vqai=hGuQ8kuc9pgc9s8qqaq=dirpe0xb9q8qiLsFr0=vr0=vr0dc8meaabaqaciaacaGaaeqabaqabeGadaaakeaafaqabeGabaaabaGaemOuaiLaeyypa0Jaemizaq2aaWbaaSqabeaacqaIYaGmaaGccqaHYoGydaWgaaWcbaGaeeOzaygabeaakiabek7aInaaBaaaleaacqqGTbqBaeqaaOGaeyOeI0YaaSGaaeaacqaIXaqmaeaacqaIYaGmaaGaeqOSdi2aaSbaaSqaaiabb2gaTbqabaGccqGGOaakcqGGOaakcqWGHbqycqGHsislcqWGKbazcqGGPaqkcqWGWbaCcqGGOaakcqWGbbqqdaWgaaWcbaGaeeOzaygabeaakiabcMcaPiabgUcaRiabcIcaOiabdggaHjabgUcaRiabdsgaKjabcMcaPiabdchaWjabcIcaOiabdkeacnaaBaaaleaacqqGMbGzaeqaaOGaeiykaKIaeiykaKYaaWbaaSqabeaacqaIYaGmaaGccqGHsisldaWccaqaaiabigdaXaqaaiabikdaYaaacqaHYoGydaWgaaWcbaGaeeOzaygabeaakiabcIcaOiabcIcaOiabdggaHjabgkHiTiabdsgaKjabcMcaPiabdchaWjabcIcaOiabdgeabnaaBaaaleaacqqGTbqBaeqaaOGaeiykaKIaey4kaSIaeiikaGIaemyyaeMaey4kaSIaemizaqMaeiykaKIaemiCaaNaeiikaGIaeqOSdi2aaSbaaSqaaiabb2gaTbqabaGccqGGPaqkcqGGPaqkdaahaaWcbeqaaiabikdaYaaaaOqaaiabgUcaRiabikdaYiabes8a0jabcUfaBnaaliaabaGaeGymaedabaGaeGinaqdaaiabcIcaOiabcIcaOiabdggaHjabgkHiTiabdsgaKjabcMcaPiabcIcaOiabdchaWjabcIcaOiabdgeabnaaBaaaleaacqqGMbGzaeqaaOGaeiykaKIaey4kaSIaemiCaaNaeiikaGIaemyqae0aaSbaaSqaaiabb2gaTbqabaGccqGGPaqkcqGGPaqkcqGHRaWkcqGGOaakcqWGHbqycqGHRaWkcqWGKbazcqGGPaqkcqGGOaakcqWGWbaCcqGGOaakcqWGcbGqdaWgaaWcbaGaeeOzaygabeaakiabcMcaPiabgUcaRiabdchaWjabcIcaOiabdkeacnaaBaaaleaacqqGTbqBaeqaaOGaeiykaKIaeiykaKIaeiykaKYaaWbaaSqabeaacqaIYaGmaaGccqGHRaWkcqWGKbazdaahaaWcbeqaaiabikdaYaaakiabcIcaOiabdchaWjabcIcaOiabdgeabnaaBaaaleaacqqGMbGzaeqaaOGaeiykaKIaeyOeI0IaemiCaaNaeiikaGIaemyqae0aaSbaaSqaaiabb2gaTbqabaGccqGGPaqkcqGGPaqkdaahaaWcbeqaaiabikdaYaaakiabc2faDjabgkHiTiabisda0iabdsgaKnaaCaaaleqabaGaeGOmaidaaOGaeqiXdq3aaWbaaSqabeaacqaIYaGmaaGccqGGUaGlaaaaaa@C4A8@

Thus, we deduced the formula for covariance between hybrid sibs as the weighted sum of three (additive, dominance and residual) components:

Cov(X1j,X2j|π⌢Mj)=f(π⌢Mj,θ) A+g(π⌢Mj,θ)  D+R.
 MathType@MTEF@5@5@+=feaafiart1ev1aaatCvAUfKttLearuWrP9MDH5MBPbIqV92AaeXatLxBI9gBamXvP5wqSXMqHnxAJn0BKvguHDwzZbqegyvzYrwyUfgarqqtubsr4rNCHbGeaGqiA8vkIkVAFgIELiFeLkFeLk=iY=Hhbbf9v8qqaqFr0xc9pk0xbba9q8WqFfeaY=biLkVcLq=JHqVepeea0=as0db9vqpepesP0xe9Fve9Fve9GapdbaqaaeGacaGaaiaabeqaamqadiabaaGcbaGaee4qamKaee4Ba8MaeeODayNaeiikaGIaemiwaG1aaSbaaSqaaiabigdaXiabbQgaQbqabaGccqGGSaalcqWGybawdaWgaaWcbaGaeGOmaiJaeeOAaOgabeaakiabcYha8naaIaaabaGaeqiWdahacaGLImcadaWgaaWcbaGaeeyta0KaeeOAaOgabeaakiabcMcaPiabg2da9GWabiaa=zgacqGGOaakdaGiaaqaaiabec8aWbGaayPiJaWaaSbaaSqaaiabb2eanjabbQgaQbqabaGccqGGSaalcqaH4oqCcqGGPaqkcqqGGaaicqqGbbqqcqGHRaWkcaWFNbGaeiikaGYaaicaaeaacqaHapaCaiaawkYiamaaBaaaleaacqqGnbqtcqqGQbGAaeqaaOGaeiilaWIaeqiUdeNaeiykaKIaaGPaVlaaykW7cqqGebarcqGHRaWkcqqGsbGucqGGUaGlaaa@71E7@

One can conclude that trait covariance depends on the necessary set of parameters {*a*, *d*, *p*_1_, *p*_2_, θ}.

### Criterion for the definition of QTL position

To localize a QTL on a chromosome the maximum likelihood method was used. This method enables to choose the most suitable genetic model, estimate the modelling parameters and define the position of the QTL with the required accuracy. Note that if there are no genetic effects (*a *= 0 and *d *= 0), it is impossible to localize a QTL since the recombination fractions between the QTL and markers can not be estimated. Let us consider two genotyped markers flanking the QTL and construct the suitable log-likelihood function:

ln*L *= *const *- 1/2 ∑[ln|**V**| + (***X ***- ***E***_***X***_) **V**^-1 ^(***X ***- ***E***_***X***_)^T^],

where the summation is over the two flanking markers; ***X ***and ***E***_***X ***_are horizontal vectors of quantitative trait values and their expectations, respectively; **V **is a covariance matrix with the elements Cov(X1j,X2j|π⌢Mj)
 MathType@MTEF@5@5@+=feaafiart1ev1aaatCvAUfKttLearuWrP9MDH5MBPbIqV92AaeXatLxBI9gBaebbnrfifHhDYfgasaacH8akY=wiFfYdH8Gipec8Eeeu0xXdbba9frFj0=OqFfea0dXdd9vqai=hGuQ8kuc9pgc9s8qqaq=dirpe0xb9q8qiLsFr0=vr0=vr0dc8meaabaqaciaacaGaaeqabaqabeGadaaakeaacqqGdbWqcqqGVbWBcqqG2bGDcqGGOaakcqWGybawdaWgaaWcbaGaeGymaeJaeeOAaOgabeaakiabcYcaSiabdIfaynaaBaaaleaacqaIYaGmcqqGQbGAaeqaaOGaeiiFaW3aaicaaeaacqaHapaCaiaawkYiamaaBaaaleaacqqGnbqtcqqGQbGAaeqaaOGaeiykaKcaaa@418B@. The log-likelihood function does not change a form at multiple analyses, because to localize the QTL among multiple markers, it is necessary to test each chromosome fragment bracketed by only two adjacent genotyped markers.

We constructed the statistics as a double likelihood ratio, 2(ln*L*_1_-ln*L*_0_), where *L*_0 _is the maximum likelihood under a null hypothesis H_0_, obtained by imposing restrictions on certain parameters of interest, and *L*_1 _is the maximum likelihood under an alternative hypothesis H_1_, where these restrictions are removed. Here, we have chosen hypothesis H_1 _in which the parameter θ is not fixed, and hypothesis H_0 _in which the parameter θ is equal to the fixed value, θ_*k*_. One can let the recombination frequency between one of the markers and the QTL, θ_*k*_, be correlated with genetic distance, *k*, by the Kocambi mapping function [27], and take into account interference:

θ_*k *_= 1/2 (e^4*k *^- 1)/(e^4*k *^+ 1),

where *k *varies from 0 to *r *discretely (with given step length), and *r *is a fixed genetic distance between two markers. Thus, we have several null hypotheses from which it is necessary to choose a suitable one. If the value of the statistics is calculated for each probable *k*-position of the QTL and compared with the critical value, then we can accept or reject the given position as correct. Indeed, the specified criterion is the linkage test, for which the critical value transformed from LOD score is equal to 2ln(10^3^) = 13.8. Note that in spite of the fact that many authors have demonstrated that, for evidence of more significant linkage, LOD score threshold is greater than 3, we use just this traditional threshold as being more convenient for comparison of our method with other ones with same LOD score thresholds. But researchers can choose a more severe threshold.

### Power

From mathematical statistics it is known that the likelihood ratio test has a central χ^2^-distribution under a null hypothesis and a noncentral χ^2^-distribution under an alternative hypothesis in large samples [25]. Given a critical *P*-value, the power of a χ^2^-test can be determined from the noncentrality parameter, *λ*, which is directly proportional to the sample size, *N*, and to the degree of freedom of the noncentral χ^2^-distribution, *df*. To estimate the power for any sample size at a given *λ *and *df*, one can refer to the appropriate function of the noncentral χ^2^-distribution. It is possible to derive analytical formulas for the noncentrality parameter without carrying out data simulation [28]. For this, it is necessary to obtain the asymptotical values of the maximum-likelihood estimates of parameters under both the H_0 _and H_1 _hypotheses, and then to take the log-likelihood expectations under these hypotheses evaluated at their respective asymptotical parameter estimations. The noncentrality parameter is then:

λ = E(2ln*L*_1_) - E(2ln*L*_0_).

The linkage test is caused by distinctions only in covariance matrixes, **V**, according to the marker IBD-distribution. For example, we constructed a noncentrality parameter for sibs since they have identical variance components. For notational convenience, we assume that the quantitative trait has unit variance, so that V_A_, V_D_, V_R _and V_e _represent both the variances and the proportions of variance. Then under the H_0 _hypothesis, when *k *varies from 0 to *r*, the asymptotic estimations of covariance are:

V0(i,j)={VA+VD+VR+Ve,if i=j,f(π⌢M,θ=θk)VA+g(π⌢M,θ=θk)VD+VR,if i≠j,
 MathType@MTEF@5@5@+=feaafiart1ev1aaatCvAUfKttLearuWrP9MDH5MBPbIqV92AaeXatLxBI9gBaebbnrfifHhDYfgasaacH8akY=wiFfYdH8Gipec8Eeeu0xXdbba9frFj0=OqFfea0dXdd9vqai=hGuQ8kuc9pgc9s8qqaq=dirpe0xb9q8qiLsFr0=vr0=vr0dc8meaabaqaciaacaGaaeqabaqabeGadaaakeaacqqGwbGvdaWgaaWcbaGaeGimaadabeaakiabcIcaOiabbMgaPjabcYcaSiabbQgaQjabcMcaPiabg2da9maaceqabaqbaeqabiqaaaqaaiabbAfawnaaBaaaleaacqqGbbqqaeqaaOGaey4kaSIaeeOvay1aaSbaaSqaaiabbseaebqabaGccqGHRaWkcqqGwbGvdaWgaaWcbaGaeeOuaifabeaakiabgUcaRiabbAfawnaaBaaaleaacqqGLbqzaeqaaOGaeiilaWIaeeyAaKMaeeOzayMaeeiiaaIaeeyAaKMaeyypa0JaeeOAaOMaeiilaWcabaacbeGae8NzayMaeiikaGYaaicaaeaacqaHapaCaiaawkYiamaaBaaaleaacqqGnbqtaeqaaOGaeiilaWIaeqiUdeNaeyypa0JaeqiUde3aaSbaaSqaaiabdUgaRbqabaGccqGGPaqkcqqGwbGvdaWgaaWcbaGaeeyqaeeabeaakiabgUcaRiab=DgaNjabcIcaOmaaIaaabaGaeqiWdahacaGLImcadaWgaaWcbaGaeeyta0eabeaakiabcYcaSiabeI7aXjabg2da9iabeI7aXnaaBaaaleaacqWGRbWAaeqaaOGaeiykaKIaeeOvay1aaSbaaSqaaiabbseaebqabaGccqGHRaWkcqqGwbGvdaWgaaWcbaGaeeOuaifabeaakiabcYcaSiabbMgaPjabbAgaMjabbccaGiabbMgaPjabgcMi5kabbQgaQjabcYcaSaaaaiaawUhaaaaa@7B40@

and under the alternative H_1 _hypothesis, when θ is not fixed, they are:

V1(i,j)={VA+VD+VR+Ve,if i=j,f(π⌢M,θ)VA+g(π⌢M,θ)VD+VR,if i≠j,
 MathType@MTEF@5@5@+=feaafiart1ev1aaatCvAUfKttLearuWrP9MDH5MBPbIqV92AaeXatLxBI9gBaebbnrfifHhDYfgasaacH8akY=wiFfYdH8Gipec8Eeeu0xXdbba9frFj0=OqFfea0dXdd9vqai=hGuQ8kuc9pgc9s8qqaq=dirpe0xb9q8qiLsFr0=vr0=vr0dc8meaabaqaciaacaGaaeqabaqabeGadaaakeaacqqGwbGvdaWgaaWcbaGaeGymaedabeaakiabcIcaOiabbMgaPjabcYcaSiabbQgaQjabcMcaPiabg2da9maaceqabaqbaeqabiqaaaqaaiabbAfawnaaBaaaleaacqqGbbqqaeqaaOGaey4kaSIaeeOvay1aaSbaaSqaaiabbseaebqabaGccqGHRaWkcqqGwbGvdaWgaaWcbaGaeeOuaifabeaakiabgUcaRiabbAfawnaaBaaaleaacqqGLbqzaeqaaOGaeiilaWIaeeyAaKMaeeOzayMaeeiiaaIaeeyAaKMaeyypa0JaeeOAaOMaeiilaWcabaacbeGae8NzayMaeiikaGYaaicaaeaacqaHapaCaiaawkYiamaaBaaaleaacqqGnbqtaeqaaOGaeiilaWIaeqiUdeNaeiykaKIaeeOvay1aaSbaaSqaaiabbgeabbqabaGccqGHRaWkcqWFNbWzcqGGOaakdaGiaaqaaiabec8aWbGaayPiJaWaaSbaaSqaaiabb2eanbqabaGccqGGSaalcqaH4oqCcqGGPaqkcqqGwbGvdaWgaaWcbaGaeeiraqeabeaakiabgUcaRiabbAfawnaaBaaaleaacqqGsbGuaeqaaOGaeiilaWIaeeyAaKMaeeOzayMaeeiiaaIaeeyAaKMaeyiyIKRaeeOAaOMaeiilaWcaaaGaay5Eaaaaaa@72A0@

In the presence of marker information, E(2ln*L*_*k*_), from formula (10) for both hypotheses (*k *= 0 or 1) is calculated as [28]:

E(2ln⁡Lk)=−E(ln⁡|Vk|)−E(X−EX)Vk−1(X−EX)T=−∑ipiln⁡|Vki|−s,
 MathType@MTEF@5@5@+=feaafiart1ev1aaatCvAUfKttLearuWrP9MDH5MBPbIqV92AaeXatLxBI9gBaebbnrfifHhDYfgasaacH8akY=wiFfYdH8Gipec8Eeeu0xXdbba9frFj0=OqFfea0dXdd9vqai=hGuQ8kuc9pgc9s8qqaq=dirpe0xb9q8qiLsFr0=vr0=vr0dc8meaabaqaciaacaGaaeqabaqabeGadaaakeaafaqabeGabaaabaGaeeyrauKaeiikaGIaeGOmaiJagiiBaWMaeiOBa4MaemitaW0aaSbaaSqaaiabdUgaRbqabaGccqGGPaqkcqGH9aqpcqGHsislcqqGfbqrcqGGOaakcyGGSbaBcqGGUbGBcqGG8baFieqacqWFwbGvdaWgaaWcbaGaee4AaSgabeaakiabcYha8jabcMcaPiabgkHiTiabbweafjabcIcaOGqadiab+HfayjabgkHiTiab+veafnaaBaaaleaacqGFybawaeqaaOGaeiykaKIae8Nvay1aa0baaSqaaiabbUgaRbqaaiabgkHiTiabigdaXaaakiabcIcaOiab+HfayjabgkHiTiab+veafnaaBaaaleaacqGFybawaeqaaOGaeiykaKYaaWbaaSqabeaacqqGubavaaaakeaacqGH9aqpcqGHsisldaaeqaqaaiabbchaWnaaBaaaleaacqqGPbqAaeqaaOGagiiBaWMaeiOBa4MaeiiFaWNae8Nvay1aaSbaaSqaaiabbUgaRjabbMgaPbqabaGccqGG8baFcqGHsislcqWGZbWCcqGGSaalaSqaaiabbMgaPbqab0GaeyyeIuoaaaaaaa@6D20@

where *s *is sibship size; and p_i _and **V**_ki _are the probability and the covariance matrix for the *i*th marker genotype configuration, respectively.

We defined the noncentrality parameter for the sib-pair and then generalized it for an entire sibship. For any relative pair, one can unambiguously assign three covariance matrices z_π _differing by a portion of IBD-alleles on the QTL, π = 0, 1/2 or 1. From formula (9) it follows that their non-diagonal elements are equal to z_π = 0 _(1,2) = V_R_, z_π = 0,5 _(1,2) = 1/2 V_A _+ V_R _and z_π = 1 _(1,2) = V_A _+ V_D _+ V_R_, and diagonal elements are obviously equal to 1. In a random sample of sib-pairs, these covariance matrices are expected to occur in the proportions 1/4:1/2:1/4, so that the noncentrality parameter for sibship is:

λ=E(ln⁡|V0|)−E(ln⁡|V1|)=14ln⁡|V0,π=0|+12ln⁡|V0,π=0,5|+14ln⁡|V0,π=1|−14ln⁡|V1,π=0|−12ln⁡|V1,π=0,5|−14ln⁡|V1,π=1|.
 MathType@MTEF@5@5@+=feaafiart1ev1aaatCvAUfKttLearuWrP9MDH5MBPbIqV92AaeXatLxBI9gBaebbnrfifHhDYfgasaacH8akY=wiFfYdH8Gipec8Eeeu0xXdbba9frFj0=OqFfea0dXdd9vqai=hGuQ8kuc9pgc9s8qqaq=dirpe0xb9q8qiLsFr0=vr0=vr0dc8meaabaqaciaacaGaaeqabaqabeGadaaakeaafaqabeGabaaabaGaeq4UdWMaeyypa0JaeeyrauKaeiikaGIagiiBaWMaeiOBa4MaeiiFaWNaeeOvay1aaSbaaSqaaiabicdaWaqabaGccqGG8baFcqGGPaqkcqGHsislcqqGfbqrcqGGOaakcyGGSbaBcqGGUbGBcqGG8baFcqqGwbGvdaWgaaWcbaGaeGymaedabeaakiabcYha8jabcMcaPaqaaiabg2da9maaliaabaGaeGymaedabaGaeGinaqdaaiGbcYgaSjabc6gaUjabcYha8jabbAfawnaaBaaaleaacqaIWaamcqGGSaalcqaHapaCcqGH9aqpcqaIWaamaeqaaOGaeiiFaWNaey4kaSYaaSGaaeaacqaIXaqmaeaacqaIYaGmaaGagiiBaWMaeiOBa4MaeiiFaWNaeeOvay1aaSbaaSqaaiabicdaWiabcYcaSiabec8aWjabg2da9iabicdaWiabcYcaSiabiwda1aqabaGccqGG8baFcqGHRaWkdaWccaqaaiabigdaXaqaaiabisda0aaacyGGSbaBcqGGUbGBcqGG8baFcqqGwbGvdaWgaaWcbaGaeGimaaJaeiilaWIaeqiWdaNaeyypa0JaeGymaedabeaakiabcYha8jabgkHiTmaaliaabaGaeGymaedabaGaeGinaqdaaiGbcYgaSjabc6gaUjabcYha8jabbAfawnaaBaaaleaacqaIXaqmcqGGSaalcqaHapaCcqGH9aqpcqaIWaamaeqaaOGaeiiFaWNaeyOeI0YaaSGaaeaacqaIXaqmaeaacqaIYaGmaaGagiiBaWMaeiOBa4MaeiiFaWNaeeOvay1aaSbaaSqaaiabigdaXiabcYcaSiabec8aWjabg2da9iabicdaWiabcYcaSiabiwda1aqabaGccqGG8baFcqGHsisldaWccaqaaiabigdaXaqaaiabisda0aaacyGGSbaBcqGGUbGBcqGG8baFcqqGwbGvdaWgaaWcbaGaeGymaeJaeiilaWIaeqiWdaNaeyypa0JaeGymaedabeaakiabcYha8jabc6caUaaaaaa@A82D@

The conditional sib-pair correlations of trait values, given the IBD-status at the QTL, can be deduced from the conditional distribution Pr(π_QTLj_|π_Mj_) as:

c0=VR+(1−Ψ)VA+(1−Ψ)2VD,c1=VR+12VA+Ψ(1−Ψ)VD,c2=VR+ΨVA+Ψ2VD,
 MathType@MTEF@5@5@+=feaafiart1ev1aaatCvAUfKttLearuWrP9MDH5MBPbIqV92AaeXatLxBI9gBaebbnrfifHhDYfgasaacH8akY=wiFfYdH8Gipec8Eeeu0xXdbba9frFj0=OqFfea0dXdd9vqai=hGuQ8kuc9pgc9s8qqaq=dirpe0xb9q8qiLsFr0=vr0=vr0dc8meaabaqaciaacaGaaeqabaqabeGadaaakeaafaqaaeWabaaabaGaee4yam2aaSbaaSqaaiabicdaWaqabaGccqGH9aqpcqqGwbGvdaWgaaWcbaGaeeOuaifabeaakiabgUcaRiabcIcaOiabigdaXiabgkHiTiabfI6azjabcMcaPiabbAfawnaaBaaaleaacqqGbbqqaeqaaOGaey4kaSIaeiikaGIaeGymaeJaeyOeI0IaeuiQdKLaeiykaKYaaWbaaSqabeaacqaIYaGmaaGccqqGwbGvdaWgaaWcbaGaeeiraqeabeaakiabcYcaSaqaaiabbogaJnaaBaaaleaacqaIXaqmaeqaaOGaeyypa0JaeeOvay1aaSbaaSqaaiabbkfasbqabaGccqGHRaWkdaWccaqaaiabigdaXaqaaiabikdaYaaacqqGwbGvdaWgaaWcbaGaeeyqaeeabeaakiabgUcaRiabfI6azjabcIcaOiabigdaXiabgkHiTiabfI6azjabcMcaPiabbAfawnaaBaaaleaacqqGebaraeqaaOGaeiilaWcabaGaee4yam2aaSbaaSqaaiabikdaYaqabaGccqGH9aqpcqqGwbGvdaWgaaWcbaGaeeOuaifabeaakiabgUcaRiabfI6azjabbAfawnaaBaaaleaacqqGbbqqaeqaaOGaey4kaSIaeuiQdK1aaWbaaSqabeaacqaIYaGmaaGccqqGwbGvdaWgaaWcbaGaeeiraqeabeaakiabcYcaSaaaaaa@6DD8@

where Ψ = θ^2 ^+ (1-θ)^2^. The noncentrality parameter for sib-pair is then given by:

λ = 1/4 ln(1-c_0_^2^) + 1/2 ln(1-c_1_^2^) + 1/4 ln(1-c_2_^2^) - 1/4 ln(1-c'_0_^2^) - 1/2 ln(1-c'_1_^2^) - 1/4 ln(1-c'_2_^2^),

where c'_i _are values of c_i _(*i *= 0, 1, and 2) at the fixed Ψ_k_, Ψ_k _= θ_k_^2 ^+ (1-θ_k_)^2^.

When the first-order approximation, ln(1-*x*)≈-*x*, is used for small values of *x*, the formula for the noncentrality parameter becomes the simplest:

λ1≈14(c'0 2−c0 2)+12(c'1 2−c1 2)+14(c'2 2−c2 2)=Δ1Δ2{12VA 2+VDVA+12(1+Δ1 2+Δ2 2)VD 2},
 MathType@MTEF@5@5@+=feaafiart1ev1aaatCvAUfKttLearuWrP9MDH5MBPbIqV92AaeXatLxBI9gBamXvP5wqSXMqHnxAJn0BKvguHDwzZbqegyvzYrwyUfgarqqtubsr4rNCHbGeaGqiA8vkIkVAFgIELiFeLkFeLk=iY=Hhbbf9v8qqaqFr0xc9pk0xbba9q8WqFfeaY=biLkVcLq=JHqVepeea0=as0db9vqpepesP0xe9Fve9Fve9GapdbaqaaeGacaGaaiaabeqaamqadiabaaGcbaqbaeaabiqaaaqaaiabeU7aSnaaBaaaleaacqaIXaqmaeqaaOGaeyisIS7aaSGaaeaacqaIXaqmaeaacqaI0aanaaGaeiikaGIaee4yamMaei4jaCYaa0baaSqaaiabbcdaWaqaaiabbccaGiabbkdaYaaakiabgkHiTiabbogaJnaaDaaaleaacqqGWaamaeaacqqGGaaicqqGYaGmaaGccqGGPaqkcqGHRaWkdaWccaqaaiabigdaXaqaaiabikdaYaaacqGGOaakcqqGJbWycqGGNaWjdaqhaaWcbaGaeeymaedabaGaeeiiaaIaeeOmaidaaOGaeyOeI0Iaee4yam2aa0baaSqaaiabbgdaXaqaaiabbccaGiabbkdaYaaakiabcMcaPiabgUcaRmaaliaabaGaeGymaedabaGaeGinaqdaaiabcIcaOiabbogaJjabcEcaNmaaDaaaleaacqqGYaGmaeaacqqGGaaicqqGYaGmaaGccqGHsislcqqGJbWydaqhaaWcbaGaeeOmaidabaGaeeiiaaIaeeOmaidaaOGaeiykaKcabaGaeyypa0JaeuiLdq0aaSbaaSqaaiabigdaXaqabaGccqqHuoardaWgaaWcbaGaeGOmaidabeaakiabcUha7naaliaabaGaeGymaedabaGaeGOmaidaaiabbAfawnaaDaaaleaacqqGbbqqaeaacqqGGaaicqqGYaGmaaGccqGHRaWkcqqGwbGvdaWgaaWcbaGaeeiraqeabeaakiabbAfawnaaBaaaleaacqqGbbqqaeqaaOGaey4kaSYaaSGaaeaacqaIXaqmaeaacqaIYaGmaaGaeiikaGIaeGymaeJaey4kaSIaeuiLdq0aa0baaSqaaiabbgdaXaqaaiabbccaGiabbkdaYaaakiabgUcaRiabfs5aenaaDaaaleaacqqGYaGmaeaacqqGGaaicqqGYaGmaaGccqGGPaqkcqqGwbGvdaqhaaWcbaGaeeiraqeabaGaeeiiaaIaeeOmaidaaOGaeiyFa0NaeiilaWcaaaaa@9601@

where Δ_1 _= Ψ-Ψ_k _and Δ_2 _= 1-Ψ_k_-Ψ. In this case λ_1 _is proportional only to the squares and products of the additive V_A _and dominance V_D _variance components and does not depend on the component conditioned by the hybrid (inter-population) origin of the sibs.

For a sufficiently accurate calculation of the noncentrality parameter, as often as not the second-order approximation, ln(1*-x*)≈-*x*-1/2 *x*^2^, is used. It follows therefore that

λ ≈ λ_1 _+ 1/8(c'_0_^4 ^- c_0_^4^) + 1/4(c'_1_^4 ^- c_1_^4^) + 1/8(c'_2_^4 ^- c_2_^4^).

The analytical expression for the noncentrality parameter after substituting the expressions (11) in formula (12) is lengthy, but we can see that *λ *depends on all variance components, V_A_, V_D_, and V_R_. Moreover, the power to detect a given QTL effect increases with increasing proportion of the residual component, V_R_. To obtain more accurate results, it is possible to use an approximation by involving higher-order terms.

To determine a noncentrality parameter for the entire sibship, we used a suitable approximation to calculate a determinant of correlation matrix (non-singular and symmetric) as shown in [28]:

ln|**V**| ≈ ln(1 - ∑V_jk_^2^) = -∑V_jk_^2^,

where ∑ denotes the sum over all possible sib-pairs (*j*, *k*), *j *<*k*. Then for an *s*-size sibship the noncentrality parameter, λ_*s*_, is equal to:

λ_s _≈ 1/2 *s*(*s *- 1) λ,

where 1/2*s*(*s*-1) is the number of sib-pairs. As is obvious, the noncentrality parameter for the linkage test is proportional to the number of all pairs in the sibship. It is noteworthy that formula (14) is not exact for small samples, and in this case, it is necessary to calculate the power through data simulation.

In the case of analyses of a hybrid pedigree of arbitrary structure, the noncentrality parameter can be obtained in a similar manner. For this purpose, noncentrality parameters are calculated for all relative pairs of the pedigree analysed, and are then summarized according to approximation (13). When the theoretical noncentrality parameter has been obtained, it is easy to calculate the size of the sample required for any required level of significance and power. For the linkage test, the level of significance required is traditionally set at a LOD score of 3, which is equivalent to a χ^2 ^statistics of 13.8 with *df *= 2 and a fixed-sample one-tailed significance level of 0.0001. The noncentrality parameter required for 80% power is 20.8 [28]. For example, the number of sib-pairs required can be obtained by dividing the noncentrality parameter required (i.e. 20.8 for the linkage test) by the theoretical noncentrality parameter per sib-pair.

Table 6 demonstrates the sample sizes required for 80% power at the critical value of 13,8 for a range of variance components, V_R_, and Ψ. Additive and dominance components are assumed to be 0.15. Sham et al. [28] have shown that detection of QTL by linkage is only feasible if the proportion of QTL variance considered is 10% or more. At this level of QTL variance, more than 20000 sib-pairs are required for linkage analysis.

**Table 6:** Sample sizes required for 80% power to detect linkage for the range of V_R_, recombination fraction and fixed components V_D _= 0.15 and V_A _= 0.15

Recombination fraction, θ	Sample size required for V_R _=							
	
	0.00	0.05	0.10	0.15	0.20	0.25	0.30	0.35
0.00	1530	1460	1379	1291	1200	1110	1023	940
0.05	2453	2346	2220	2082	1938	1793	1653	1520
0.10	4078	3909	3704	3478	3240	3001	2767	2545
0.15	7142	6855	6504	6111	5697	5279	4871	4480

### Simulations

To examine the performance of the proposed approach in realistic situations we conducted simulation studies on examples of inbred sibships. We generated 10, 20, 30, and 40 hybrid pedigrees covering three generations of individuals (*F*_0_, *F*_1_, and *F*_2 _generations). Founders (*F*_0 _individuals) of each pedigree analysed are two individuals from the *P*_1 _population and single individual from the *P*_2 _population. The founders from different populations formed a crossing pair and had one offspring for the *F*_1 _generation. Inbred crossing between related *F*_1 _individuals contributed to the *F*_2 _generation by the size of 10 offsprings. Thus, each pedigree consisted of three founders, two *F*_1 _individuals, and ten *F*_2 _individuals. For all inbred sibs from the *F*_2 _generation, the marker genotypes and phenotypic values of the trait were simulated and were considered as known.

We considered two QTL positions between two markers, in turn assuming that the QTL is located in the chromosome positions 5 and 10 cM, and that the markers flanking it are fixed at positions 0 and 25 cM. We specified the distribution of allele frequencies of the QTL for all the individuals analysed based on the assumption that the Hardy-Weinberg equilibrium is carried out for the founders.

Let there be four-allelic markers (two unique alleles from each initial population). The marker genotypes of the founders were selected randomly, assuming an even distribution of frequencies of marker alleles. The additive and dominance genetic values were taken to be *a *= 3 and *d *= 1, respectively. For the founders, the QTL allelic frequencies were taken to be *p*_1 _= 0.9 and *p*_2 _= 0.4. The phenotypic values were obtained by adding the normal deviation *N*(0,1) to the genetic value. For each given set of model parameters {*p*_1_, *p*_2_, *a*, *d*, θ} and the given sample size, 100 replicates were simulated.

Our purpose is to locate the QTL estimating allelic frequencies for initial populations *P*_1 _and *P*_2 _and the genetic effects of the trait in question in each replicate. To locate the QTL on a chromosome fragment between markers, we discretely moved along the fragment at a step length of 0.01 cM and estimated the double likelihood ratio statistics at each point. If the statistics calculated at a point was higher than the critical value, then the hypothesis of the localization of the QTL at this point was rejected. The QTL was hypothetically located at the point where the statistics had the lowest value.

We compared our method with the method for QTL analysis of *F*_2 _crosses between outbred lines, described in [2,29], which was performed using the Qxpak software available free at [30].

The performance of both methods was tested using the same simulated data. The comparative characteristics were the frequency of the events consisting in the fact that the true location of the QTL would not rejected (W_1_), and the frequency of the events consisting in the fact that the statistical test would indicate the true QTL location as the most likely one (W_2_). It is obvious that W_1 _≥ W_2_. It should be noted that the value of (1-W_1_) can be interpreted as type I error rate, and value of W_2 _can be analogous to power of the method.

**Figure 2:**
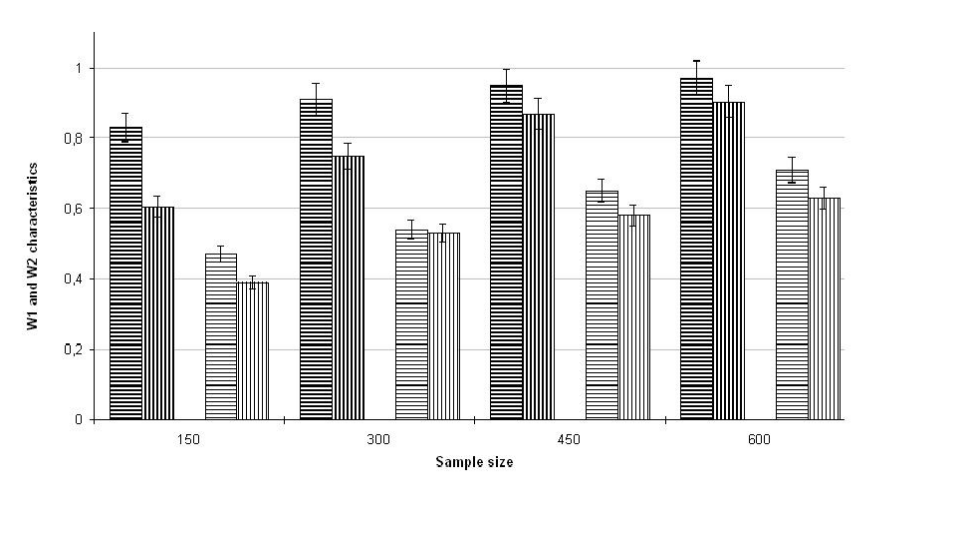
**Bar graphs for W_1 _and W_2 _statistical characteristics depending on the sample size at the QTL position 5 cM**. Bar graphs hatched by bold lines correspond to characteristic W_1_; bar graphs hatched by thin  lines correspond to characteristic W_2_; vertical lines are intended for the method realized by software Qxpak; horizontal lines are intended for the method proposed in this study.

**Figure 3:**
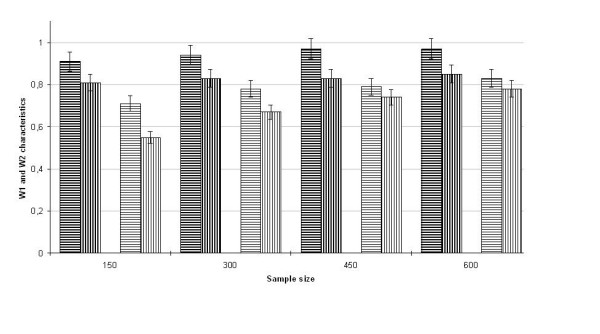
**Bar graphs for W_1 _and W_2 _statistical characteristics depending on the sample size at the QTL position 10 cM**. Bar graphs hatched by bold lines correspond to characteristic W_1_; bar graphs hatched by thin lines correspond to characteristic W_2_; vertical lines are intended for the method realized by software Qxpak; horizontal lines are intended for the method proposed in this study.

We compared the W_1 _and W_2 _frequencies for the two methods at different sample sizes, *N*_ped _= 150, 300, 450, and 600. Figures 2 and 3 show the results of simulation studies for two positions of the QTL, 5 and 10 cM, respectively. As can be seen, at both positions of the QTL, the statistical characteristics W_1 _and W_2 _calculated by the method proposed are higher than those calculated using Qxpak at all sample sizes considered.

When the QTL is really localized at the position 5 cM (Figure 2), our method demonstrates the highest values of W_1 _frequency, exceeding 0.8 at any sample size. Only beginning at *N*_ped _= 450, each of bar graphs for W_1 _gets in a range of 5-percentage error of another. Regarding the W_2 _characteristic, its values do not exceed 0.8 for either method at any sample size. When the QTL is localized at position 10 cM (Figure 3), the frequencies W_1 _and W_2 _are higher than 0.95 and 0.8, respectively, beginning at *N*_ped _= 150 for our method and beginning at *N*_ped _> 600 for Qxpak.

All aforesaid facts speak in favour for our method for the analysis of hybrid pedigrees with dominance and inbreeding effects. The results obtained justify the QTL analysis by our method that yields more accurate data on the localization of the QTL.

## Discussion

In this study we have updated the variance-components method for the analysis of hybrid pedigrees with dominance and inbreeding. We have considered hybrid sibships as an example to demonstrate the method. An advantage of our method is to partition into variance components, where one of the components is conditioned by the inter-population origin of individuals and inbreeding. There is no necessity to resolve this component into separate elements caused by inter-population origin and separate elements caused by inbreeding since these elements are indivisible in variances and covariances and therefore can not be estimated singly.

We have derived an intuitively appealing result regarding the power of our method under a variance-components model for larger samples of sibships. If the effects of QTL are small, the results are particularly simple. We have generally arrived at the conclusion that the power of our method decreases rapidly with decreasing proportion of the variance component caused by the hybrid origin and by inbreeding. This means that the sample size required for 80% power for hybrid pedigrees is less than for pedigrees descended from one pure line.

For simplicity, we did not consider such fixed factors affecting the formation of traits as for example, age and sex, but these factors can easily be included in our model. Moreover, the method suggested can be used to choose the most suitable model for the description of the data: additive models (*d *= 0), dominance models (*a *= 0), models of crosses of two pure lines (*p*_1 _= 0, and *p*_2 _= 1) or models of intra-population crosses (*p*_1_=*p*_2_).

The results obtained make it possible to draw conclusions on the competence of the incorporated analysis that could specify not only the localization of the QTL, but also an estimate of the values of QTL effects.

## Conclusion

We have presented a new modification of the variance-components method for QTL analysis. It is a linkage test method, whose originality consists in considering the trait effect caused by inter-population origin and inbreeding. Analytical derivations for the variance components make it possible to analyse their dependence from the model parameters.

The analytical expressions for the power of our method avoid the intensive computations required for simulated data processing and allow to estimate the size of the pedigree required. We have shown that the method is more powerful if the QTL effects conditioned by inter-population origin and inbreeding are increased. Several improvements can be developed to take into account fixed factors affecting trait formation, such as age and sex.

Our method uses the trait values and the marker information for each individual of a pedigree with an arbitrary structure including inbred loops as initial data and can be valuable for fine mapping purposes.

## Methods

To choose the most correct genetic model for quantitative trait formation, estimate the modelling parameters, and define the position of the QTL on a chromosome with the accuracy required, we employed statistics based on likelihood maximization:

max⁡a,d,p1,p2ln⁡L(a,d,p1,p2,θ|π⌢Mj)
 MathType@MTEF@5@5@+=feaafiart1ev1aaatCvAUfKttLearuWrP9MDH5MBPbIqV92AaeXatLxBI9gBaebbnrfifHhDYfgasaacH8akY=wiFfYdH8Gipec8Eeeu0xXdbba9frFj0=OqFfea0dXdd9vqai=hGuQ8kuc9pgc9s8qqaq=dirpe0xb9q8qiLsFr0=vr0=vr0dc8meaabaqaciaacaGaaeqabaqabeGadaaakeaadaWfqaqaaiGbc2gaTjabcggaHjabcIha4bWcbaGaemyyaeMaeiilaWIaemizaqMaeiilaWIaemiCaa3aaSbaaWqaaiabigdaXaqabaWccqGGSaalcqWGWbaCdaWgaaadbaGaeGOmaidabeaaaSqabaGccyGGSbaBcqGGUbGBcqWGmbatcqGGOaakcqWGHbqycqGGSaalcqWGKbazcqGGSaalcqWGWbaCdaWgaaWcbaGaeGymaedabeaakiabcYcaSiabdchaWnaaBaaaleaacqaIYaGmaeqaaOGaeiilaWIaeqiUdeNaeiiFaW3aaicaaeaacqaHapaCaiaawkYiamaaBaaaleaacqqGnbqtcqqGQbGAaeqaaOGaeiykaKcaaa@54F2@

This maximization is numerically carried out using the simplex METHI – program specifically developed to obtain maximum likelihood (ML) and ML-parameter estimates of likelihood function. METHI uses a method of configurations when maximising a function. We have free access to METHGI on our laboratory website [31]. The parameters {*a*, *d*, *p*_1_, *p*_2_} must be estimated. For the recombination frequency, we have assigned different fixed values corresponding to specific distances on the chromosome.

## Appendix

Let the relationship degree between the father (*i*_f_) and the mother (*i*_m_) of an inbred individual (*i*) be equal to (1/2)^k^, and the inbreeding parameter, τ_k_, be calculated by formula (3). Using the mathematical induction method, we show that τ_k+1 _= 1/2τ_k_, if a single non-inbred (*k*+1)-relative is to be inserted into the inbred loop. For certainty, we added the new member between individuals *i *and *i*_f_. Consequently, we re-denoted the *i*th individual as the *j*th individual because his genotype distribution had changed. Apparently, the renamed *j*th individual has the former mother *j*_m _= *i*_m_, and his father is the added relative denoted as *j*_f_. In one turn, the father of the new member is the father of the *i*th individual *j*_ff _= *i*_f_, and the mother of the new member is the individual *j*_fm_, unrelated to the father. It is easy to find the frequency of genotype *AA *of the offspring *j *analysed at the outbred cross:

p(AAj)=p(Ajm)p(Ajf)=p(Ajm)(p(Aif)+p(Aifm))/2,
 MathType@MTEF@5@5@+=feaafiart1ev1aaatCvAUfKttLearuWrP9MDH5MBPbIqV92AaeXatLxBI9gBaebbnrfifHhDYfgasaacH8akY=wiFfYdH8Gipec8Eeeu0xXdbba9frFj0=OqFfea0dXdd9vqai=hGuQ8kuc9pgc9s8qqaq=dirpe0xb9q8qiLsFr0=vr0=vr0dc8meaabaqaciaacaGaaeqabaqabeGadaaakeaafaqadeGabaaabaGaemiCaaNaeiikaGIaemyqaeKaemyqae0aaSbaaSqaaiabbQgaQbqabaGccqGGPaqkcqGH9aqpcqWGWbaCcqGGOaakcqWGbbqqdaWgaaWcbaGaeeOAaOMaeeyBa0gabeaakiabcMcaPiabdchaWjabcIcaOiabdgeabnaaBaaaleaacqqGQbGAcqqGMbGzaeqaaOGaeiykaKcabaGaeyypa0JaemiCaaNaeiikaGIaemyqae0aaSbaaSqaaiabbQgaQjabb2gaTbqabaGccqGGPaqkcqGGOaakcqWGWbaCcqGGOaakcqWGbbqqdaWgaaWcbaGaeeyAaKMaeeOzaygabeaakiabcMcaPiabgUcaRiabdchaWjabcIcaOiabdgeabnaaBaaaleaacqqGPbqAcqqGMbGzcqqGTbqBaeqaaOGaeiykaKIaeiykaKIaei4la8IaeGOmaiJaeiilaWcaaaaa@5F80@

and at the inbred cross:

pinb(AAj)=∑Gop(AAj|Go)p(Go)=∑Gop(Aim|Go)p(Ajf|Go)p(Go),
 MathType@MTEF@5@5@+=feaafiart1ev1aaatCvAUfKttLearuWrP9MDH5MBPbIqV92AaeXatLxBI9gBaebbnrfifHhDYfgasaacH8akY=wiFfYdH8Gipec8Eeeu0xXdbba9frFj0=OqFfea0dXdd9vqai=hGuQ8kuc9pgc9s8qqaq=dirpe0xb9q8qiLsFr0=vr0=vr0dc8meaabaqaciaacaGaaeqabaqabeGadaaakeaafaqadeGabaaabaGaemiCaa3aaWbaaSqabeaacqqGPbqAcqqGUbGBcqqGIbGyaaGccqGGOaakcqWGbbqqcqWGbbqqdaWgaaWcbaGaeeOAaOgabeaakiabcMcaPiabg2da9maaqababaGaemiCaaNaeiikaGIaemyqaeKaemyqae0aaSbaaSqaaiabbQgaQbqabaGccqGG8baFcqWGhbWrdaWgaaWcbaGaee4Ba8gabeaakiabcMcaPiabdchaWjabcIcaOiabdEeahnaaBaaaleaacqqGVbWBaeqaaOGaeiykaKcaleaacqWGhbWrdaWgaaadbaGaee4Ba8gabeaaaSqab0GaeyyeIuoaaOqaaiabg2da9maaqababaGaemiCaaNaeiikaGIaemyqae0aaSbaaSqaaiabbMgaPjabb2gaTbqabaGccqGG8baFcqWGhbWrdaWgaaWcbaGaee4Ba8gabeaakiabcMcaPiabdchaWjabcIcaOiabdgeabnaaBaaaleaacqqGQbGAcqqGMbGzaeqaaOGaeiiFaWNaem4raC0aaSbaaSqaaiabb+gaVbqabaGccqGGPaqkcqWGWbaCcqGGOaakcqWGhbWrdaWgaaWcbaGaee4Ba8gabeaakiabcMcaPiabcYcaSaWcbaGaem4raC0aaSbaaWqaaiabb+gaVbqabaaaleqaniabggHiLdaaaaaa@70F1@

where *G*_o _designates the set of genotypes of the common ancestors for parents of the *j*th individual. We expressed the frequencies of genotypes of the father *j*_f _through frequencies of his parents:

pinb(AAj)=12∑Gop(Aim|Go)[p(Ajff|Go)+p(Ajfm)]p(Go).=12∑Gop(Aim|Go)[p(Aif|Go)+p(Ajfm)]p(Go).
 MathType@MTEF@5@5@+=feaafiart1ev1aaatCvAUfKttLearuWrP9MDH5MBPbIqV92AaeXatLxBI9gBaebbnrfifHhDYfgasaacH8akY=wiFfYdH8Gipec8Eeeu0xXdbba9frFj0=OqFfea0dXdd9vqai=hGuQ8kuc9pgc9s8qqaq=dirpe0xb9q8qiLsFr0=vr0=vr0dc8meaabaqaciaacaGaaeqabaqabeGadaaakeaafaqadeGabaaabaGaemiCaa3aaWbaaSqabeaacqqGPbqAcqqGUbGBcqqGIbGyaaGccqGGOaakcqWGbbqqcqWGbbqqdaWgaaWcbaGaeeOAaOgabeaakiabcMcaPiabg2da9maaliaabaGaeGymaedabaGaeGOmaidaamaaqababaGaemiCaaNaeiikaGIaemyqae0aaSbaaSqaaiabbMgaPjabb2gaTbqabaGccqGG8baFcqWGhbWrdaWgaaWcbaGaee4Ba8gabeaakiabcMcaPiabcUfaBjabdchaWjabcIcaOiabdgeabnaaBaaaleaacqqGQbGAcqqGMbGzcqqGMbGzaeqaaOGaeiiFaWNaem4raC0aaSbaaSqaaiabb+gaVbqabaGccqGGPaqkcqGHRaWkcqWGWbaCcqGGOaakcqWGbbqqdaWgaaWcbaGaeeOAaOMaeeOzayMaeeyBa0gabeaakiabcMcaPiabc2faDjabdchaWjabcIcaOiabdEeahnaaBaaaleaacqqGVbWBaeqaaOGaeiykaKIaeiOla4caleaacqWGhbWrdaWgaaadbaGaee4Ba8gabeaaaSqab0GaeyyeIuoaaOqaaiabg2da9maaliaabaGaeGymaedabaGaeGOmaidaamaaqababaGaemiCaaNaeiikaGIaemyqae0aaSbaaSqaaiabbMgaPjabb2gaTbqabaGccqGG8baFcqWGhbWrdaWgaaWcbaGaee4Ba8gabeaakiabcMcaPiabcUfaBjabdchaWjabcIcaOiabdgeabnaaBaaaleaacqqGPbqAcqqGMbGzaeqaaOGaeiiFaWNaem4raC0aaSbaaSqaaiabb+gaVbqabaGccqGGPaqkcqGHRaWkcqWGWbaCcqGGOaakcqWGbbqqdaWgaaWcbaGaeeOAaOMaeeOzayMaeeyBa0gabeaakiabcMcaPiabc2faDjabdchaWjabcIcaOiabdEeahnaaBaaaleaacqqGVbWBaeqaaOGaeiykaKIaeiOla4caleaacqWGhbWrdaWgaaadbaGaee4Ba8gabeaaaSqab0GaeyyeIuoaaaaaaa@9A3C@

After of some transformations, it is clear that the frequency of genotype *AA *at the inbred cross can be expressed through a similar frequency of the individual *i*th:

*p*^inb^(*AA*_j_) = 1/2(*p*^inb^(*AA*_i_) + *p*(*A*_jfm_) *p*(*A*_im_)).

The inbreeding parameter τ_k+1 _is then:

τk+1=pinb(AAj)−p(AAj)=12(pinb(AAi)+p(Ajfm)p(Aim)−p(Ajm)[p(Aif)+p(Aifm)])=12(pinb(AAi)−p(AAi))=12τk.
 MathType@MTEF@5@5@+=feaafiart1ev1aaatCvAUfKttLearuWrP9MDH5MBPbIqV92AaeXatLxBI9gBaebbnrfifHhDYfgasaacH8akY=wiFfYdH8Gipec8Eeeu0xXdbba9frFj0=OqFfea0dXdd9vqai=hGuQ8kuc9pgc9s8qqaq=dirpe0xb9q8qiLsFr0=vr0=vr0dc8meaabaqaciaacaGaaeqabaqabeGadaaakeaafaqadeabbaaaaeaacqaHepaDdaWgaaWcbaGaee4AaSMaey4kaSIaeGymaedabeaakiabg2da9iabdchaWnaaCaaaleqabaGaeeyAaKMaeeOBa4MaeeOyaigaaOGaeiikaGIaemyqaeKaemyqae0aaSbaaSqaaiabbQgaQbqabaGccqGGPaqkcqGHsislcqWGWbaCcqGGOaakcqWGbbqqcqWGbbqqdaWgaaWcbaGaeeOAaOgabeaakiabcMcaPaqaaiabg2da9maaliaabaGaeGymaedabaGaeGOmaidaaiabcIcaOiabdchaWnaaCaaaleqabaGaeeyAaKMaeeOBa4MaeeOyaigaaOGaeiikaGIaemyqaeKaemyqae0aaSbaaSqaaiabbMgaPbqabaGccqGGPaqkcqGHRaWkcqWGWbaCcqGGOaakcqWGbbqqdaWgaaWcbaGaeeOAaOMaeeOzayMaeeyBa0gabeaakiabcMcaPiabdchaWjabcIcaOiabdgeabnaaBaaaleaacqqGPbqAcqqGTbqBaeqaaOGaeiykaKIaeyOeI0IaemiCaaNaeiikaGIaemyqae0aaSbaaSqaaiabbQgaQjabb2gaTbqabaGccqGGPaqkcqGGBbWwcqWGWbaCcqGGOaakcqWGbbqqdaWgaaWcbaGaeeyAaKMaeeOzaygabeaakiabcMcaPiabgUcaRiabdchaWjabcIcaOiabdgeabnaaBaaaleaacqqGPbqAcqqGMbGzcqqGTbqBaeqaaOGaeiykaKIaeiyxa0LaeiykaKcabaGaeyypa0ZaaSGaaeaacqaIXaqmaeaacqaIYaGmaaGaeiikaGIaemiCaa3aaWbaaSqabeaacqqGPbqAcqqGUbGBcqqGIbGyaaGccqGGOaakcqWGbbqqcqWGbbqqdaWgaaWcbaGaeeyAaKgabeaakiabcMcaPiabgkHiTiabdchaWjabcIcaOiabdgeabjabdgeabnaaBaaaleaacqqGPbqAaeqaaOGaeiykaKIaeiykaKcabaGaeyypa0ZaaSGaaeaacqaIXaqmaeaacqaIYaGmaaGaeqiXdq3aaSbaaSqaaiabbUgaRbqabaGccqGGUaGlaaaaaa@9F10@

Thus, we have shown that the inbreeding parameter for the individual descended from any type of inbred cross, depends on the degree of relationship of his parents and genotype distribution of the common ancestors, and does not depend on the distribution of the inbred offspring.

As a result, inbreeding changes the parameters of distribution of quantitative trait values for hybrid individuals: genotypic means decrease or are constant, and covariances basically increase. It is important to note that the account of inbreeding of hybrid individuals does not complicate QTL analysis, and more exactly estimates parameters of distribution of quantitative trait values.
